# Contrasting Patterns of Single Nucleotide Polymorphisms and Structural Variation Across Multiple Invasions

**DOI:** 10.1093/molbev/msad046

**Published:** 2023-02-23

**Authors:** Katarina C Stuart, Richard J Edwards, William B Sherwin, Lee A Rollins

**Affiliations:** Evolution & Ecology Research Centre, School of Biological, Earth and Environmental Sciences, UNSW Sydney, Sydney, New South Wales, Australia; School of Biological Sciences, University of Auckland, Auckland, New Zealand; Evolution & Ecology Research Centre, School of Biotechnology and Biomolecular Sciences, UNSW Sydney, Sydney, New South Wales, Australia; Evolution & Ecology Research Centre, School of Biological, Earth and Environmental Sciences, UNSW Sydney, Sydney, New South Wales, Australia; Evolution & Ecology Research Centre, School of Biological, Earth and Environmental Sciences, UNSW Sydney, Sydney, New South Wales, Australia

**Keywords:** *sturnus vulgaris*, invasive species, common starling, balancing selection, directional selection

## Abstract

Genetic divergence is the fundamental process that drives evolution and ultimately speciation. Structural variants (SVs) are large-scale genomic differences within a species or population and can cause functionally important phenotypic differences. Characterizing SVs across invasive species will fill knowledge gaps regarding how patterns of genetic diversity and genetic architecture shape rapid adaptation under new selection regimes. Here, we seek to understand patterns in genetic diversity within the globally invasive European starling, *Sturnus vulgaris*. Using whole genome sequencing of eight native United Kingdom (UK), eight invasive North America (NA), and 33 invasive Australian (AU) starlings, we examine patterns in genome-wide SNPs and SVs between populations and within Australia. Our findings detail the landscape of standing genetic variation across recently diverged continental populations of this invasive avian. We demonstrate that patterns of genetic diversity estimated from SVs do not necessarily reflect relative patterns from SNP data, either when considering patterns of diversity along the length of the organism's chromosomes (owing to enrichment of SVs in subtelomeric repeat regions), or interpopulation diversity patterns (possibly a result of altered selection regimes or introduction history). Finally, we find that levels of balancing selection within the native range differ across SNP and SV of different classes and outlier classifications. Overall, our results demonstrate that the processes that shape allelic diversity within populations is complex and support the need for further investigation of SVs across a range of taxa to better understand correlations between often well-studied SNP diversity and that of SVs.

## Introduction

Genetic divergence is the fundamental process that drives evolution and ultimately speciation (Wu 2001). This genetic variation results from a complex interplay of factors influencing patterns of genetic variation that can be selectively neutral (e.g., demographic effects such as drift or range-edge effects, Robertson 1962) and/or adaptive (e.g., selection, spatial sorting, Schluter 2000; Arnold et al. 2001). Much remains unknown about the interacting roles of genetic diversity, drift, gene flow, and selection in determining evolutionary trajectories (Campbell et al. 2018). Further, these evolutionary changes occasionally occur over very short biological timescales, a phenomenon termed rapid adaptation or rapid evolution (Thompson 1998). Central to this are questions about how genetic variants arise and remain within a population without being deleterious to individual fitness (Agrawal and Whitlock 2012), and conversely how these genetic variants may respond to selective regimes (Kimura 1991; Eyre-Walker and Keightley 2007).

One major class of genetic mutations is structural variants (SVs), which includes deletions (DEL), insertions (INS), duplications (DUP), inversions (INV), and translocations (TRA) (Alkan et al. 2011; Alonge et al. 2020). SVs are large-scale mutations within an organism's genome that can have a profound impact on the phenotype. Due to their size, single SVs have the potential to introduce more substantial phenotypic changes than single nucleotide polymorphisms (SNPs), as they can encompass multiple functional genetic elements (Alkan et al. 2011; Alonge et al. 2020). SVs may directly alter gene content (Zhao et al. 2020) or affect transcription levels of adjacent genes (Puig et al. 2004). Examples of dramatic SV-affected phenotypes include altered pathogen resistance (Hämälä et al. 2021), modified plant flowering times (Todesco et al. 2020), and reproductive strategies (Lamichhaney et al. 2016).

The study of SVs has been somewhat limited until recent years, both due to their previously supposed rarity as well as the computational difficulties in characterizing them (Ho et al. 2020). A range of bioinformatic processes now exists through which people may characterize structural variants across a variety of sequencing data types including short-read, long-read, and RNA-seq (Mahmoud et al. 2019; Ho et al. 2020). These technical developments are allowing the role SVs play in adaptation and speciation to be examined, and also provide an opportunity to contrast neutral and adaptive patterns in population genetic structure and diversity using SNP and SV data across a wide range of taxa. Within humans, SNPs explain less than a tenth of the between-individual genetic variation when considering all genetic variants, including SVs (Pang et al. 2010). Within the marine teleost *Chrysophrys auratus,* SV coverage of the genome was threefold that of SNPs (Catanach et al. 2019), and within chocolate trees, SV coverage is eight times that of SNPs (Hämälä et al. 2021). While a direct comparison of SV and SNP numbers across studies may be difficult due to different sequencing and data processing approaches, it is likely that SVs may play an important role in adaptation, with their effects being unable to be captured by SNP analysis alone (Dorant et al. 2020). It is expected that SNP-derived patterns of genetic diversity will be largely reflected in SV data because many SNPs are tightly associated with SVs. Nevertheless, a study of SVs alongside SNPs allows for a more comprehensive quantification of diversity and may reveal novel characteristics of adaptive divergence within a population (Catanach et al. 2019). Understanding the role SVs may play in genetic divergence is hence of vital importance for understand standing genetic variation and the adaptive capacity of a population.

The importance of understanding patterns in SVs extends to invasive species, where they serve as a potential mechanism for the rapid evolution that is often seen in invasive ranges (Colautti and Barrett 2013; Vandepitte et al. 2014; Rollins et al. 2015; Stapley et al. 2015). Characterizing SVs across invasive species will help fill in knowledge gaps regarding patterns of genetic diversity and genetic architecture within successful invasive populations (Bock et al. 2015). Studies of SVs that try to explain a phenotype difference between organisms (e.g., finding a large explanatory inversion to explain phenotypic differences: Todesco et al. 2020) need to be complemented with an understanding of the range of SV types and their frequencies over a diverse range of different populations (e.g., Mérot et al. 2020), and studies of this range are scarce. Comparing different types of genetic variants will allow us to determine if SV patterns reflect that of SNPs within a population and will also shed light on the underlying mechanisms of evolution.

**Fig. 1.**
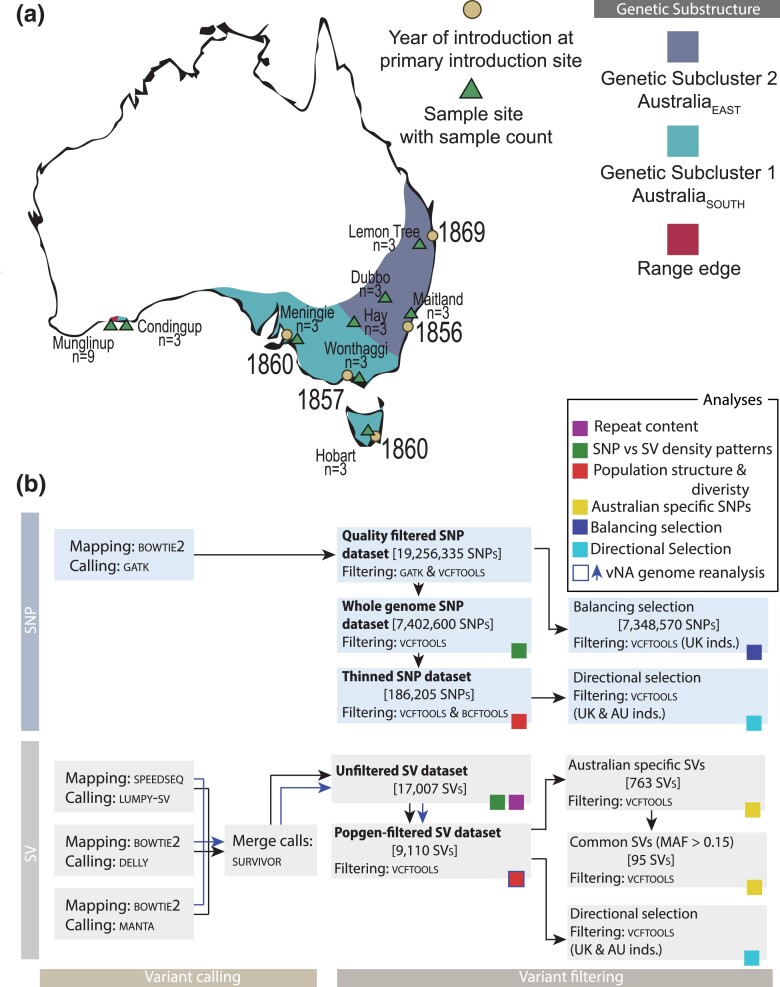
Summary of the sample sites within the starling's (*Sturnus vulgaris*) AU range, and variant calling and filtering used in various analysis. (*a*) Starling's AU range (based on eBird data retrieved in 2018), with genetic substructuring indicated by the two large genetic subclusters AU_EAST_ (along the east coast of Australia, encompasing Lemon Tree, Dubbo, and Maitland, with Hay shared across both subclusters) and AU_SOUTH_ (along the south coast of Australia, emcompassing Wonthaggi, Hobart, Meningie, and Condingup, with Hay shared across both subclusters), as well as the range edge (Munglinup). Primary introduction sites and sample sites are indicated by circles and triangles respectively. In addition to this, we also had eight samples each from UK and NA. (*b*) Bold names of datasets in the “filtering” section are named and referred to directly in the manuscript text. Additional datasets generated by extra filtering choices are also listed in this figure (see methods for rationales and justifications).

The globally invasive European starling (*Sturnus vulgaris*) is native to the Palearctic and was introduced to Australia and North America in 1857 and 1890, respectively. Within Australia, starlings were introduced to multiple sites, with historical records indicating most introduced individuals came from the United Kingdom (Jenkins 1977). Range expansion from introduction sites has resulted in a fairly continuous range that has strong substructure differentiating the eastern and southern halves of the range (fig. 1*a*, Rollins et al. 2009, 2011; Stuart et al. 2021). This genetic differentiation is likely due to founder effects, as well as drift and selection happening independently following introduction and during subsequent range expansion (Stuart et al. 2021; Stuart, Cardilini et al. 2022). Microsatellite, mitochondrial, and reduced representation sequencing have also confirmed strong genetic differentiation at the western-most range edge (Munglinup, Western Australia), thought to be a result of rapid adaptation and/or an extreme genetic bottleneck (Rollins et al. 2009, 2011; Stuart et al. 2021).

In this manuscript, we seek to understand differences in patterns of genome-wide SNPs and SVs within the globally invasive European starling. We used whole genome sequencing of eight native range starlings from the United Kingdom (UK), eight invasive North American starlings (NA), and 33 invasive Australian (AU) starlings to examine patterns in genome-wide SNPs and SVs both between native and invasive populations, and also within the highly structured AU population. Specifically, we aimed to assess overall SV patterns in starlings globally and compare the population genetic patterns of SNPs and SVs across the three starling populations. We then investigated signatures of SNP and SV adaptation between invasive AU and the native UK to assess whether variants diverging between the populations result from regional differences in balancing and directional selection, or have been maintained as neutral variants. We predicted that balancing selection helps to maintain standing genetic variation (de Filippo et al. 2016; Huang et al. 2016; Koenig et al. 2019; Stern and Lee 2020) for SNPs and SVs within the native range (UK) onto which directional selection may act following introduction.

## Materials and Methods

### SNP Genetic Sequencing Data

The whole genome resequencing short-read Illumina data used in this project focused primarily on the AU range to allow for a thorough study of adaptive and neutral genetic patterns within this highly structured and well-characterized population. The eight sampling sites (*n* = 3 individuals each) were chosen either for their proximity to primary introduction sites, to capture part of the inland range, or capture the range edge of the population (fig. 1*a*). In addition to this, extra sampling (*n* = 9 individuals) was conducted in the range edge to try to resolve the origins of this genetically distinct region. In addition to these 33 AU samples, eight samples from New York, North America (NA; invasive range), and Newcastle upon Tyne, United Kingdom (UK; native range) were included in this study to provide a native range and a second invasive range comparison (for details, including which samples have been used previously, please see supplementary appendix 1, Supplementary Material online, supplementary table S1, Supplementary Material online).

**Whole genome SNP dataset.** To obtain SNP variant information, the raw reads were processed using Samtools (v1.9) (Li et al. 2009) and Bedtools (v2.27.1) (Quinlan and Hall 2010), and adapters were removed using Adapterremoval (v2.2.2) (Schubert et al. 2016). The processed reads were aligned to the starling reference genome *S. vulgaris* vAU1.0 (Stuart, Edwards et al. 2022) using Bowtie2 (v 2.3.5.1) (Langmead and Salzberg 2012), and indexed using Picard (v2.18.26) (‘Picard toolkit” 2019). The Gatk (v4.1.0.0) (Poplin et al. 2018) function *HaplotypeCaller* (GVCF mode) was used to call haplotypes for each sample, which were processed with *CombineGVCFs* and finally passed into *GenotypeGVCFs* to produce the initial VCF file (30,153,260 SNPs). The VCF file was put through an initial filter step in GATK (QD < 2.0, FS > 60.0, MQ < 40.0, SOR > 3.0), and a secondary filter using Vcftools (v0.1.16) (Danecek et al. 2011) (max proportion of missing alleles per SNP = 0.5, minor allele frequency (MAF) = 0.03, min mean DP (approximate read depth) = 2, max mean DP = 50, min alleles = 2, max alleles = 2), producing a total of 19,256,335 SNP variants. These SNPs were then pruned in Bcftools for linkage by removing sites with an *r*^2^ > 0.6 within 1000 bp site windows (Karlsson Linnér et al. 2019), resulting in 7,402,600 SNP variants. This created the variant file used in the balancing selection analysis because these analysis require high-density SNP data (Siewert and Voight 2020). Hereafter, this dataset will be referred to as the whole genome SNP dataset (fig. 1*b*).

**Thinned SNP dataset.** For all other SNP analyses high-density was not required, and having completely independent SNPs is more appropriate because it reduced overcontribution of groups of linked SNPs to analyses. We created a list of independent SNPs by thinning these variants in Vcftools, retaining only SNPs more than 5,000 bp from one another (−thin 5,000), resulting in 186,205 SNPs. This dataset was checked for sample relatedness using Vcftools (−relatedness2), which confirmed there were no full siblings within the dataset (all relatedness values <0.25). Hereafter, this SNP dataset will be referred to as the thinned SNP dataset.

### SV Calling Using Short-Read Data

We re-analyzed the above 49 short-read WGS (whole genome sequencing) sample data for SVs using three different short-read SV calling programs: Lumpy-sv v0.2.13 (Layer et al. 2014), Delly v0.8.2 (Rausch et al. 2012), and Manta v1.6.0 (Chen et al. 2016). The three chosen SV callers use read-pair and split-read data (and in the case of Lumpy-sv read depth as well) as detection signals for the presence of SVs in sample read data. Further, calling SVs across multiple programs, and then combining the individual calls into a consensus SV list based on overlap between datasets, helps to reduce the false positive rates from using a singular caller, which is of particular importance for short-read data which is more vulnerable to this type of error (Jeffares et al. 2017). For Lumpy-sv, we used the traditional pipeline; we mapped the read data using Speedseq (Chiang et al. 2015), generated empirical size histograms and statistics for each sample BAM file using Samtools (Li et al. 2009), identified SV's by running Lumpy on all samples simultaneously alongside the sample-specific histogram information, and used Svtyper (Chiang et al. 2015) to call genotypes, with max reads set to 2,000 (to remove regions of extreme coverage and decrease run time). For Delly, we followed the “germline SV calling” pipeline, using the Bowtie2 mapped reads (see Section SNP Genetic Sequencing Data). For Manta, we used the default pipeline using the Bowtie2 mapped reads (see Section SNP Genetic Sequencing Data) and processed the variant file using the Manta function *convertInversion.py* to extract INV from break end calls. For Delly and Manta, we used only those calls with the PASS filter, whereas for Lumpy-sv (which has no default filter) we kept only the calls with a minimum depth of 5 (Vcftools min-meanDP 5).

**Unfiltered SV dataset.** We then created a consensus SV file using Survivor v1.0.3 (Jeffares et al. 2017), which merges SV calls based on type and position consensus across individual SV callers. We merged the three sets of SV calls for each sample separately using the parameter values of “1000 2 1 1 0 30” (max allowed distance between predicted break ends 1 kb, minimum 2 SV caller support needed to retain a call, type, and strand of the SV must agree, and minimum SV length of 30 bp). Precise definitions vary between studies, but in this study, we retained SVs greater than 30 bp long. This created a consensus list of SV for each individual. However, Survivor does not have a built-in setting to have SV merging informed by the genotypes called within each individual SV calling program. Thus we modified the recommended pipeline so that SV calls were only retained if the minimum two SV callers also agreed on the individual's genotype (this was done by splitting each individual's Lumpy-sv, delly, and Manta VCF based on the three possible genotypes, and merging using the above-mentioned parameter values, then combining all retained SV calls). We then combined the retained reads across samples to obtain an overall SV file, using the parameters “1000 1 1 1 0 30” (same as above except minimum required support by the caller was set to 1 to produce a merged SV list). This resulted in a file containing 17,007 SVs, and hereafter will be referred to as the unfiltered SV dataset (fig. 1*b*). In all SV datasets, TRA (translocations) refers specifically to break ends.

### SV Repeat Classification and Final SV Filtering

We characterized the repeat content of the unfiltered SV dataset by using RepeatMasker v4.0.7 (Smit et al. 2013) and a custom-curated repeats database generated by Stuart, Edwards et al. (2022). We performed Repeatmasker searches on all of the SV sequences, using the first 30 bp of each SV sequence obtained by Bedtools*getfasta*. Searching the repeat database for matches to the first 30 bp of each SV focused on the repeats that existed at the break end site, and ensured long SVs did not return numerous hits from the middle of their length.

**Popgen-filtered SV dataset**. The unfiltered SV dataset was further filtered using Vcftools for missingness and MAF at the same threshold as the SNP data (−max-missing 0.5 and –maf 0.03), resulting in 9,110 SVs. Hereafter, this dataset will be referred to as the popgen-filtered SV dataset (fig. 1*b*).

### Examining SNP and SV Density Patterns

To investigate whether global patterns in SNP and SV density were correlated, Bedtools*coverage* was used to summarize SNP and SV counts (whole genome SNP dataset and unfiltered SV dataset) in 1 Mb bins along the 32 largest scaffolds of the starling genome, which was calculated for all samples, as well as within continental sample groupings (AU, NA, and UK). To assess whether SNP and SV density was correlated, the binned variant densities were analyzed alongside each other in R v3.5.3 (R Core Team 2017) using the *glm*() function (family = quasipoisson), and residuals were retained. Bedtools*intersect* was used to find SVs that overlap with coding regions, against the *S. vulgaris* vAU1.0 annotation (Stuart, Edwards et al. 2022).

To check for any batch effect or bias introduced by different total sequenced read counts in samples, the proportion of heterozygous variant sites for each individual was calculated for both SNPs and SVs (thinned SNP dataset and popgen-filtered SV dataset), and was assessed with a linear mixed model using the R function *lmer*() in the package LME4 v1.1–26 (Bates et al. 2015), with population set as a random factor. A significant result was returned for SV heterozygosity, which was not present when the sequencing batch was included as a second random effect (supplementary fig. S1, Supplementary Material online), which indicated the presence of batch effects impacting SV heterozygosity, and was taken into account in some downstream analysis.

### Comparison of Global SNP and SV Population Structuring and Diversity

To contrast patterns of population structure and genetic diversity, the following analysis was run on both the SNPs and SVs (thinned SNP dataset and popgen-filtered SV dataset). Ancestry analysis was run using Admixture (Alexander et al. 2009), while Plink v1.9 (Purcell et al. 2007) was used to assess the major pattern of variance in the data via a principal component analysis (PCA).

The program Stacks v2 function *populations* (Catchen et al. 2013) was used to quantify the number of polymorphic markers, observed heterozygosity (H_O_), expected heterozygosity (H_E_), nucleotide diversity (π), and private allele counts for both SNP and SV genetic variants (for SV we treated the alt and ref variants, regardless of type or length, the same as SNP alleles). Because of the batch effects reported above, we took a conservative approach when comparing genetic diversity statistics across populations within the main manuscript. We assessed only individuals from the Hofmeister et al. (2021) study, which includes eight individuals each from UK and NA, and eight AU individuals with four from each major subpopulation AU_EAST_ and AU_SOUTH_, all sequenced within the same batch (though we repeat this analysis, with all 49 individuals and report these results within the supplementary material, Supplementary Material online). Heterozygosity measures were assessed for statistical differences between sample sites using the R package Ggstatsplot, using the *ggwithinstats()* function for nonparametric data (Patil 2021). Analysis of SV genetic diversity was conducted twice, once on the popgen-filtered dataset, and once on a SV dataset that was recalled (using all the above steps) using the *S. vulgaris* vNA genome version (Stuart, Edwards et al. 2022), to account for possible effects of reference bias introduced by the original genome based on an AU individual.

To investigate common unique SVs that had arisen in the AU population, the popgen-filtered SV dataset was filtered to retain only AU-specific SV alleles not present in NA and UK. Munglinup individuals were excluded from this analysis (and also Section Directional and Balancing Selection) due to their extreme genetic differentiation and putatively admixed introduction history, see Section Population Genetics of *S. vulgaris* Using Whole Genome SNP and SV Data. We produced site frequency spectrum (SFS) plots for these AU-specific SVs after dividing them into two groups; those that overlapped (either entirely or partially) with a gene, and those that did not. As a point of comparison, we also produced SFS plots for the popgen-filtered SV dataset, and then subdivided this dataset into three: SVs with no coding region overlap; SVs overlapping an entire coding region; SVs with one or more break ends overlapping a coding region.

The AU-specific SVs were then further filtered to remove uncommon variants by filtering for a MAF of >0.15. We generated a list of candidate genes that exist within 1 kb of these common AU-specific SVs using Bedtools*intersect*, using the *S. vulgaris* vAU1.0 annotation (Stuart, Edwards et al. 2022).

### Directional and Balancing Selection

Adaptive changes in the frequencies of genetic variants may result from balancing selection, which enables adaptation to an intermediate or changeable environment, or from directional selection, which enables adaptation to an environment that is locally constant (Hedrick 2007). Balancing selection helps to maintain standing genetic variation within the native range and it has been hypothesized that this may provide the substrate upon which directional selection can act in invasive ranges (de Filippo et al. 2016; Huang et al. 2016; Koenig et al. 2019; Stern and Lee 2020). Here, we sought to determine if genetic variants putatively under directional selection between the native UK and invasive AU occurred in regions of higher balancing selection within the UK population.

First, we identified regions of putatively adaptive divergence in UK versus AU by using Bayescan v2.1 (Foll and Gaggiotti 2008) and Baypass v2.1 (Gautier 2015) (two programs that identify directional selection in genetic variants) analysis to identify SNP and SV genetic variants that have signals of directional selection between invasive AU and native UK (thinned SNP dataset and popgen-filtered SV dataset). We also briefly note here that the direction selection may reflect shits in either invasive AU or native UK (Stuart, Sherwin et al. 2022). We conducted Bayescan analysis with prior odds for the neutral model set to 10 for SNPs (-pr_odds 10), and 5 for SVs (-pr_odds 5, to reduce the threshold for flagged outlier SNPs so that the strongest outlier variants would lie above the statistical threshold and be available for follow-up balancing selection analysis), and a false discovery rate of 0.05. We conducted Bayescan analysis by running the core model to generate XtX statistic for the genetic variants and then calculated a significance threshold for candidate SNPs under divergent selection across sample sites through the generation of a pseudo-observed data of 5,000 SNPs, and a 1% empirical threshold was calculated for the observed XtX. To account for neutral population substructure due to demographic processes at introduction, the AU sample sites were split into two groups and the analysis was conducted on each separately. The AU sample site subdivision was based on population substructure identified in Stuart and Cardilini et al. (2021) and was also corroborated by SNP-based Admixture patterns in this dataset (see below, fig. 5*a* below, AU_EAST_ group: Lemon Tree, Maitland, Dubbo, Hay; AU_SOUTH_ group: Hay, Wonthaggi, Hobart, Meningie, Condingup; with Hay appearing twice due to ambiguous grouping, supplementary table S1, Supplementary Material online). PCA of all SNP and all SV outliers was generated using Plink v1.9 and their placement along the genome visualized using Circlize (Gu et al. 2014) in R.

To examine levels of balancing selection at genomic variants under putative directional selection, we performed genomic scans for balancing selection on the UK SNPs and SVs (separately). Scans for balancing selection on SNPs were performed with the program Betascan v2 (Siewert and Voight 2020), which uses relative allele frequencies of nearby sites to quantify a measure of balancing selection present at a loci, with high *β*^(1)^ scores being indicative of an excess of SNPs at similar frequencies within a region and thus balancing selection. Because this analysis relies on high-density of SNPs, the whole genome SNP dataset was used, and filtered to only nonfixed alleles present in UK individuals (fig. 1*b*). Further, following recommended protocols (Siewert and Voight 2020), we only retained *β*^(1)^ scores for SNPs with a minimum MAF of 0.15 to reduce false positives (Siewert and Voight 2017) because balancing selection at low allele frequencies is unlikely to overcome drift and can exaggerate the effect of drift (Robertson 1962). Because *β*^(1)^ scores are generated using high-density SNPs, SV data could not be used to directly generate *β*^(1)^ scores, nor direct *β*^(1)^ scores provided for SVs from SNP variants because SVs do not necessarily map directly to SNPs. Instead, we generated pseudo *β*^(1)^ scores for SVs by retaining all SNP *β*^(1)^ scores that occurred within 1000 bp upstream of the first break end. This approach was used because one may expect that patterns of allele frequencies may be similar for all SNPs immediately adjacent because these SNPs are expected to be linked. The value of 1,000 bp was chosen because this was the maximum allowed distance between SV calls during Survivor consensus SV merging. Then, for each SV, we obtained the trimmed mean (0.1 trimming, middle 90% data) for the *β*^(1)^ scores occurring upstream of it, to try to reduce the impact of outliers (we also tested a second approach, where *β*^(1)^ scores were not trimmed or averaged for each SV individually but were simply pooled). We used the R function *kruskal.test()* to test whether mean *β*^(1)^ scores differed across seven groups: SNPs under directional selection; SNPs not under directional selection; SVs under directional selection; and four SV types (DEL, DUP, TRA, INV) not under directional selection. INS were removed from analysis due to a sample size of 1, and SVs under directional selection were not split by SV type because all but three were DEL.

## Results

### Patterns in *S. Vulgaris* Structural Variants

**Fig. 2.**
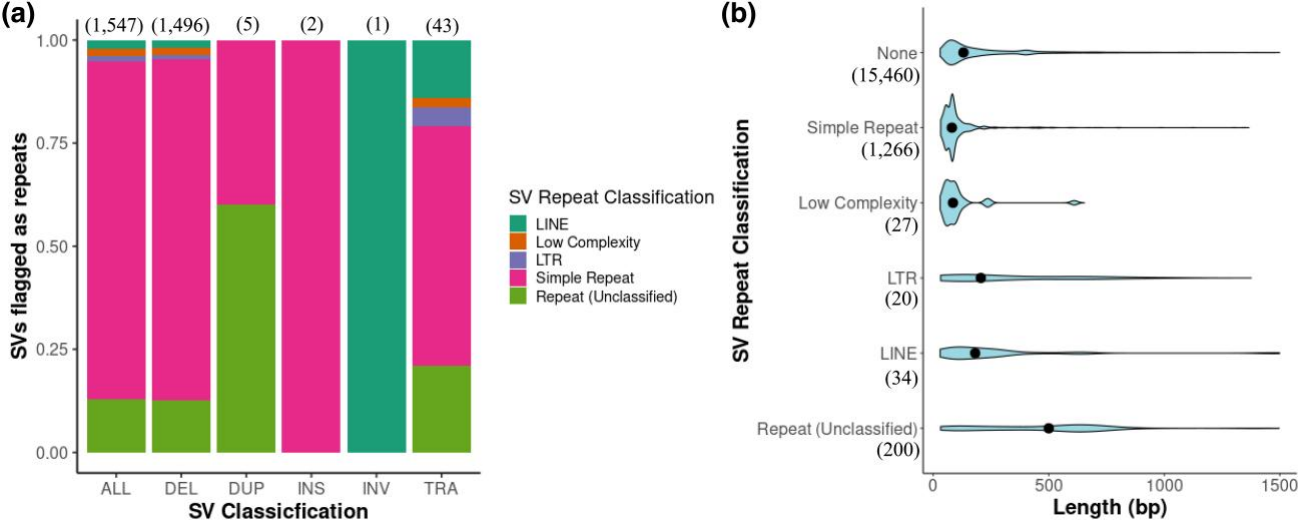
*Sturnus vulgaris* annotation of repeat elements in the unfiltered structural variant (SV) dataset across all 49 individuals. Panel (*a*) shows the proportion of repeat types across SV of different types. The total amount of SVs discovered to be repeated is indicated above each bar plot, totaling 9.10% (1,547/17,007) of the total SV count. Panel (*b*) shows length distributions of the different SV repeat classes (SVs above 1500 bp were excluded from size distribution plots).

Annotation of the unfiltered SV dataset identified repeat elements in 9.10% of the SV sequence break ends, with simple repeats making up the majority of the unfiltered repeat types (fig. 2*a*). Repeat annotation profiles for DEL and TRA, the largest of the two groups, were fairly similar, though with the latter containing a higher proportion of LINE and LTR elements (fig. 2*a*). Simple and low complexity repeats had the shortest length compared to other SV repeat classifications, while LTR and unclassified repeats (which contained a mix of species-specific repeats such as MITES) were the longest variant types (fig. 2*b*).

**Fig. 3.**
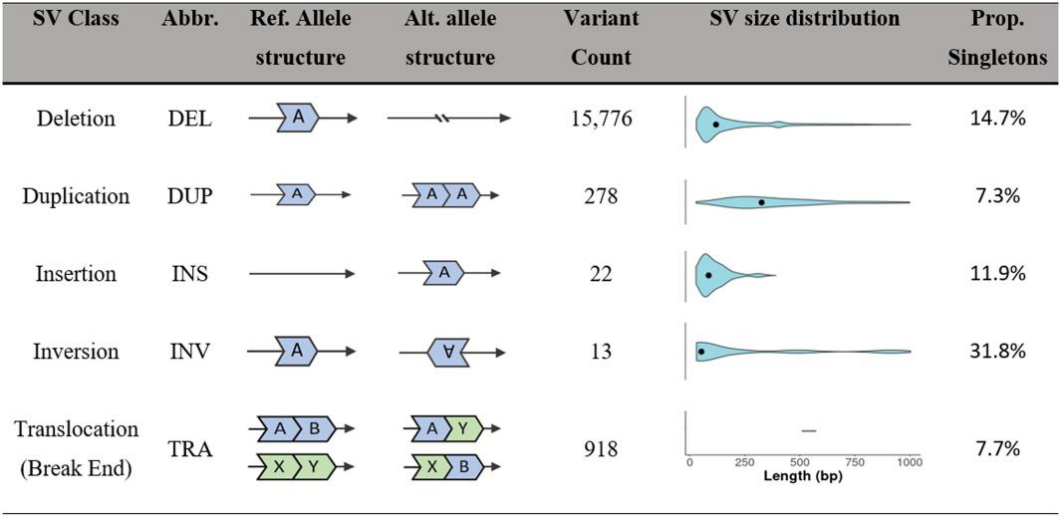
Summary of quality-filtered structural variants found across 49 *Sturnus vulgaris* individuals spread across three continents, a total of 17,007 structural variants (SVs). SV size distribution black dot indicates median length of SV class (SVs above 1000 bp were excluded from size distribution plots and median calculations). The proportion of singletons was calculated using Vcftools–singletons. SV size distribution was not calculated for TRA as these SV were called break ends, and thus quantifying size across them would be inconsistent.

Once filtered for MAF and missingness, a total of 9,110 SVs (popgen-filtered SV dataset) were identified over the 49 individuals, in comparison to the 7,402,600 unlinked SNPs identified in the whole genome SNP dataset. While the total number of SVs is much lower than that of SNPs, the total SV length (75,163,356 bp) covers 10.15 × the total length of the identified SNP variants. Across these SVs, DEL was the most common SV class, followed by TRA and DUP (fig. 3). A very small number of INV and INS were detected. Of the different SV classes, DUP was on average the largest (median length = 327) with the other SV size classes having roughly similar sizes (fig. 3; median lengths 129, 87, and 48 for DEL, INS, and INV respectively; no sizes provided for TRA because break ends have undefined sizes, medians calculated for SVs with length < 1,000). The proportion of singleton variants across all SV classes was low (31.8% or less), with DUP and TRA having the lowest (7.3% and 7.7.%, respectively; fig. 3).

**Fig. 4.**
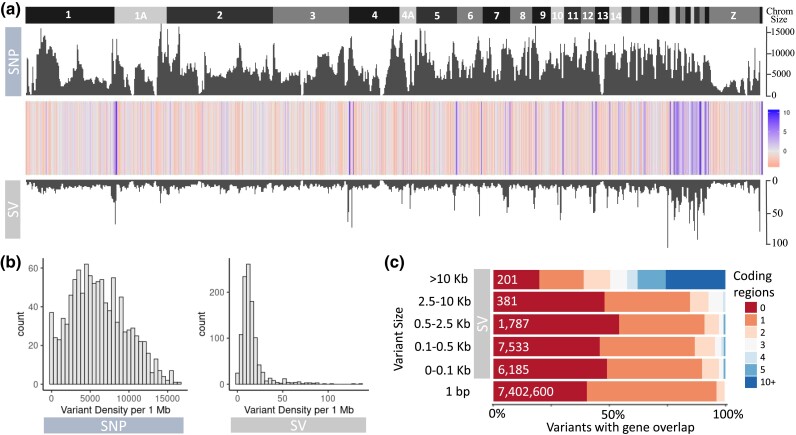
*Sturnus vulgaris* WGS single nucleotide polymorphism (SNP) and structural variant (SV) comparisons across the first 33 chromosomes. Panel (*a*) depicts the residuals from a regression of SV density on SNP density (10Mb windows) regression, with chromosome labels above the plot (positive residual corresponds to more SVs, negative corresponds to less SVs). Panel (*b*) depicts a histogram of SV and SNP densities in 1 Mb windows. Panel (*c*) depicts the proportion of each genetic variant size class that overlap with one or more genes, along with total variant count within each size classes displayed in text. Some overlapped genes in the underlying annotation have resulted in two genes flagged for 1 bp (SNP) variant size class.

The density of the SNPs and SVs were highly correlated (general linear model: *t*-value = 13.06, *P-*value < 0.0001). Visualization of residual patterns between SNPs and SVs revealed that for most of the length of the genome, SVs are underrepresented; however, there is SV enrichment near the end of chromosomes (fig. 4*a*; enrichment is also visible on smaller chromosomes, though this is likely a result of having a higher proportion of the chromosome length as chromosome ends). Overall, SVs occurred in much lower density (most commonly 16 per 1 Mb) than SNPs (most commonly 5,000 per 1 Mb) (fig. 4*b*). SNP density patterns were very similar across all three populations, whereas SV density was higher in AU compared to NA and UK (supplementary fig. S2, Supplementary Material online). Analysis of patterns of coding region coverage for variants of different sizes indicate that roughly 80–90% of variants overlapped one or no coding regions, though generally there was an increase in coding region overlap as SV size class increased (fig. 4*c*). Finally, MAF and missingness filtering was seen to reduce the number of SVs at intermediate allele frequencies (supplementary fig. S3, Supplementary Material online).

### Population Genetics of *S. Vulgaris* Using Whole Genome SNP and SV Data

**Fig. 5.**
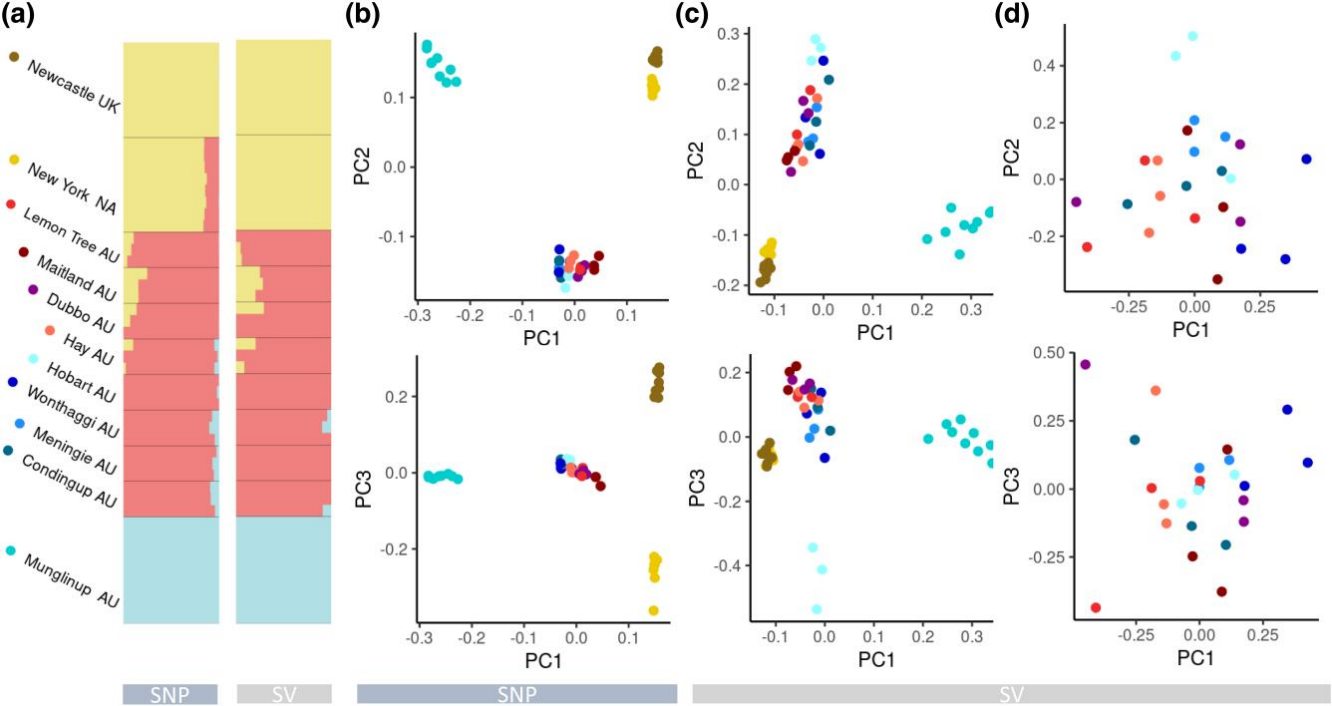
*Sturnus vulgaris* WGS single nucleotide polymorphism (SNP) and structural variant (SV) population structure and genetic diversity. Panel (*a*) contains admixture plots from SNPs (top) and SV (bottom). Panel (*b*) contains PCAs of SNP variants (thinned SNP dataset, *n* = 186,205), panel (*c*) contains PCAs of all SVs (popgen-filtered SV dataset, *n* = 9,110), and panel (*d*) contains PCA of all common AU-specific SV allele variants (*n* = 95). Colors correspond to those depicted in panel (*a*) next to each sampling site location name.

Population structure analysis conducted using Admixture on SNPs and SVs supported similar ancestry patterns across sampled populations, though SV-based population structuring contained more signals of mixed ancestry within Australia at higher K (number of ancestral subpopulations) values (fig. 5*a*, supplementary fig. S4, Supplementary Material online). AU populations contained mixed ancestry signals, with the western-most range-edge site (Munglinup) remaining distinct, and slight subpopulation structuring was identified across AU sample sites. Because the *K*-values 1–3 received similar cross-validation error support (supplementary fig. S5, Supplementary Material online), we plotted *K* = 3 as the highest *K*-value allowing us to discern more patterns, and also as means of comparing to similar analyses completed previously using some of these samples (Hofmeister et al. 2021). Similar to the Admixture analysis results, both SNP and SV PCA analyses supported distinctly separate clustering of the western range-edge, with slight substructure present in the remaining AU individuals, with AU_EAST_ (red) differentiated slightly from AU_SOUTH_ (blue) (fig. 5*b* and *c*).

Comparing genetic diversity patterns across the Hofmeister et al. (2021) subsample, we found that overall SNP genetic diversity (*π*, H_O_, H_E_) was higher in the NA and UK populations compared to AU (table 1, supplementary table S3, Supplementary Material online). However, when considering SV genetic variants, the genetic diversity across these populations was more similar to each other, or even highest in AU compared to NA and UK. While these differences are slight, many pairwise comparisons were significantly different (supplementary fig. S6–8, Supplementary Material online). This shift in the relative genetic diversities in UK, NA, and AU was still apparent even when the *S. vulgaris* vNA genome was used to call SVs (table 1). When genetic diversity analysis was expanded to all 49 samples used in this study, both SNP and SV genetic diversity patterns revealed that Munglinup contained minimal genetic diversity relative to the other AU sample sites (supplementary table S3, Supplementary Material online). Despite this reduced genetic diversity, Munglinup was found to have many private alleles (150 for the popgen-filtered SV dataset, in comparison to 60 for NA, 40 for UK, and 2–11 for remaining AU sample sites) in comparison to the Hofmeister et al. (2021) subset of a similar number of AU individuals (105 for the thinned SNP dataset, in comparison to 676 for NA, 884 for UK, and 3–10 for remaining AU sample sites).

**Table 1.** Single Nucleotide Polymorphism (SNP) and Structural Variant (SV) Summary Statistics for *Sturnus Vulgaris* Across Study Sample Sites and Continents.

Continent	Sampling Group (sample number)	Single nucleotide polymorphisms (SNP)			Structural Variants (SV)					
		Thinned SNP dataset (186,205)			*S. vulgaris* vAU genome (9,110)			*S. vulgaris* vNA genome (22,616)		
		H_o_	He	*π*	H_o_	He	*π*	H_o_	He	*π*
**Australia**	Hofmeister et al. Subset (8)	0.286	0.272	0.290	0.172	0.197	0.213	0.482	0.327	0.356
*Invasive*	AU_EAST_ (4)	0.293	0.254	0.290	0.170	0.169	0.200	0.481	0.309	0.375
	AU_SOUTH_ (4)	0.279	0.248	0.284	0.175	0.179	0.210	0.481	0.311	0.377
**North America**	New York (8)	0.311	0.289	0.308	0.159	0.173	0.187	0.479	0.323	0.352
*Invasive*										
**United Kingdom**	Newcastle upon Tyne (8)	0.315	0.295	0.314	0.163	0.175	0.189	0.486	0.327	0.355
*Native*										

H_O_ = observed heterozygosity, H_E_ = the within-population gene diversity, *π* = nucleotide diversity. H_O_, H_E_, and *π* were assessed using STACKS populations. Full information for all study sample groupings, as well as variance information, is available in supplementary table S3, Supplementary Material online. SNP dataset was the thinned SNP dataset (186,205 SNPs), and SV datasets were the popgen-filtered SV dataset (9,110 SVs), and an alternate SV dataset generated from the vNA genome version (22,616 SVs).

### AU-Specific *S. Vulgaris* SVs

**Fig. 6.**
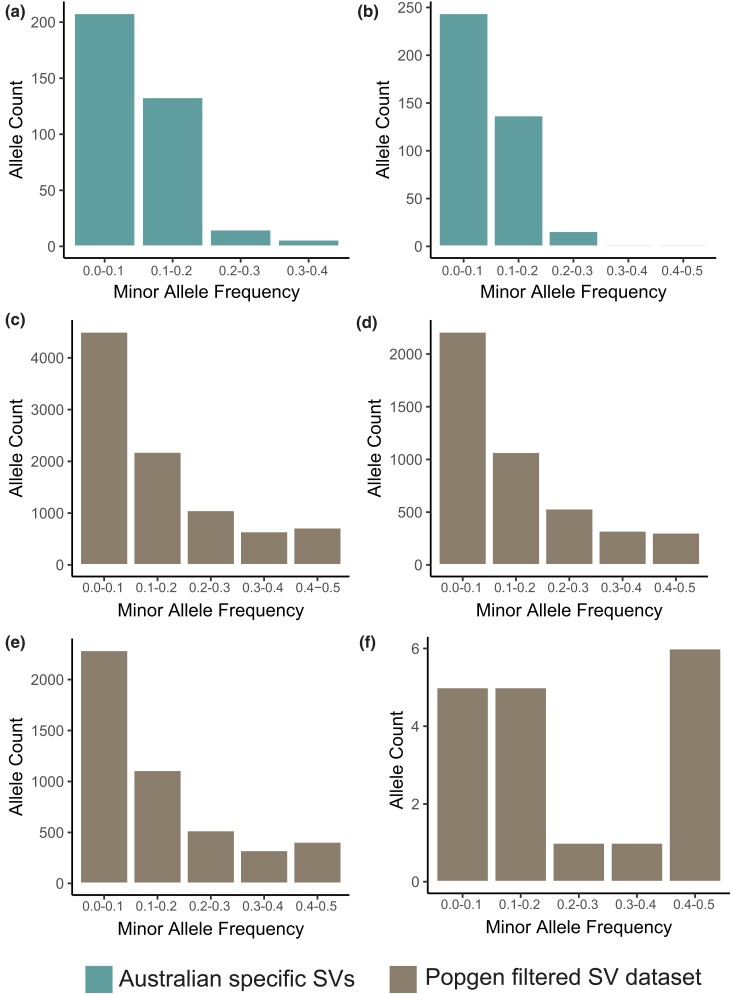
Folded site frequency spectrum (SFS) for structural variant (SV) in *Sturnus vulgaris*. Panel (*a*) and (*b*) depicts the SFS of AU-specific SVs with no coding region overlap and with (either partial or complete) coding region overlap respectively. Panel (*c*) depicts the SFS of the full popgen-filtered SV dataset, panel (*d*) the SFS of popgen-filtered SV with no coding region overlap, panel (*e*) the SFS of popgen-filtered SV with break end coding region overlap, and panel (*f*) the SFS of popgen-filtered SV with complete coding region overlap.

A total of 736 AU-specific SVs were found across the 24 AU samples (excluding the 9 Munglinup samples). The SFS of the AU-specific SVs that overlapped (either partially or completely) a coding region and those that did not were very similar (fig. 6*a* and *b*), with a vast majority of the variants falling below MAF levels of 0.1. The SFS profile of these SVs was quite different from the popgen-filtered SVs, with the latter having more common (higher MAF) alleles (fig. 6*c*). Popgen-filtered SVs that did not overlap genes (fig. 6*d*) had fewer variants at intermediate frequencies compared to SVs with break ends overlapping genes (fig. 5*e*), though this difference was slight. For popgen-filtered SV with complete coding region overlap, high MAF levels were most common (fig. 6*f*), though this group of variants was considerably rarer overall.

When the 736 AU-specific SVs were filtered for common variants (a minimum MAF of 0.15), a total of 95 variants were retained. The distinction between the two AU genetic subclusters was severely reduced when PCA analysis was conducted only on the 95 common AU-specific SV alleles (fig. 5*d*). These variants were mainly DEL (as found in the overall SV dataset) of 10,000 bp or less in length, though the largest was a DUP 316,614 bp in length. Of the variants greater than 150 bp in length, a majority were found to overlap or be within 1 kb of coding regions (supplementary table S4, Supplementary Material online). Coding regions underlying a wide range of biological processes were identified within the AU-specific SVs (supplementary table S4, Supplementary Material online, supplementary fig. S9, Supplementary Material online).

### Patterns of Directional and Balancing Selection in *S. Vulgaris* SNPs and SVs

**Fig. 7.**
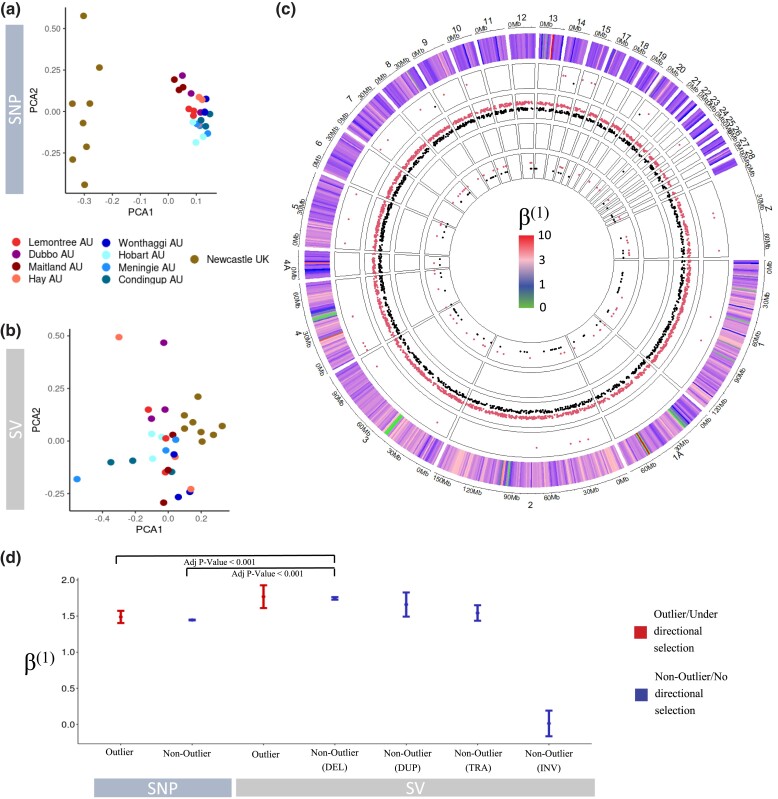
Genetic variants under putative directional selection in *Sturnus vulgaris* across the native range and invasive AU range and assessment of balancing selection, for both single nucleotide polymorphisms (SNPs) and structural variants (SVs). Panel (*a*) depicts a PCA of all outlier SNPs (pooled across the UK-AU_EAST_ and UK-AU_SOUTH_ analysis) for all AU and UK individuals. Panel (*b*) depicts a PCA of all outlier SVs (pooled across the UK-AU_EAST_ and UK-AU_SOUTH_ analysis) for all AU and UK individuals. Panel (*c*) depicts a circlize plot of mean *β*^(1)^ scores in 1 Mb windows along the genome (sex chromosome excluded), and the location of the Bayescan and Baypass outliers across four tracks (UK-AU_EAST_ in black and plotted lower within each individual track, UK-AU_SOUTH_ in red and plotted higher within each individual track) which from outside to inside are: Bayescan SNPs, Baypass SNPs, Bayescan SVs, and Baypass SVs. Panel (*d*) depicts mean *β*^(1)^ scores (balancing selection within the native UK range) for SNPs and SVs under directional selection (Bayescan and Baypass outliers between AU and UK) and no directional selection (±standard error) in UK individuals, alongside the alongside the adjusted *P*-value of Dunn's test following a significant *kruskal.test()* result (Kruskal–Wallis *χ*^2^ = 647.88, df = 6, *P*-value < 2.2 × 10*^–^*^16^). SV pseudo *β*^(1)^ scores were calculated by obtaining a trimmed mean (0.1 trimming) of overlapping/upstream SNP *β*^(1)^ scores for each SV individually, before averaging this score within each SV type group. INS was removed from analysis due to a sample size of 1.

We sought to identify genomic variants putatively under directional selection between the native UK range and invasive AU range. Out of the 186,205 SNPs tested for signatures of directional selection between AU and UK, we identified a total of 2779 SNPs (Bayescan: 3 AU_EAST_ and 33 AU_SOUTH_; Baypass: 1366 AU_EAST_ and 1825 AU_SOUTH_). Out of the 9,110 SVs tested for signatures of directional selection, we identified a total of 136 SVs (Bayescan: 0 AU_EAST_ and 1 AU_SOUTH_; Baypass: 85 AU_EAST_ and 69 AU_SOUTH_). PCA analysis of these outliers revealed much greater diversity in UK compared to AU for the outlier SNPs flagged as under putative directional selection between AU and UK (fig. 7*a*), however, the opposite was true for outlier SVs, which showed much greater diversity across AU samples (fig. 7*b*). Outlier location showed little congruence across either outlier program or variant type (fig. 7*c*).

We examined levels of balancing selection within the genomes of the UK individuals, across the genetic variants flagged as either outliers or nonoutliers. Comparing outliers (putatively under directional selection) to nonoutlier loci across SNPs and the different SV types (DEL, DUP, TRA, INV), we found a significant difference between average *β*^(1)^ scores (levels of balancing selection within the native range) in sample groupings (H_6_ = 647.88, *P*-value < 0.0001; fig. 7*d*). Post hoc analysis using Dunn's test revealed that nonoutlier and outlier SNPs had statistically lower *β*^(1)^ scores (lower levels of balancing selection in the native range) than nonoutlier SV DELs (supplementary table S5, Supplementary Material online). Using the second approach to obtaining pseudo *β*^(1)^ scores yielded similar patterns across tested SNP and SV groups, but with more significantly different pairwise comparisons (due to larger data counts) (supplementary fig. S10, Supplementary Material online, supplementary table S6, Supplementary Material online).

## Discussion

SVs are an integral component of the genetic diversity within a species, with the potential to have profound functional consequences for an organism (Puig et al. 2004; Todesco et al. 2020; Hämälä et al. 2021). Clarifying the roles that SVs play in allowing an invasive species to persist and evolve within their newly invaded range is an integral next step to understanding how genomic architecture enables invasive populations to flourish. Our findings detail the landscape of standing genetic variation across recently diverged continental populations of an invasive avian, the European starling. We demonstrate that patterns of SV genetic diversity do not necessarily reflect patterns in SNP diversity, either when considering interpopulation diversity patterns, or patterns of diversity across the genome. We also find evidence for different levels of balancing selection across SNPs and SVs, which indicated that there may be different optimum frequencies for different types of genetic variants.

### Contrasting SNP and SV Patterns Within the Genome

Broadly, patterns in SNPs and SVs were found to be significantly correlated across the three populations of starlings investigated in this study, though we identified an increase in the densities of SVs near the ends of chromosomes. This may in part be due to artifacts: for example, assembling repeat regions in reference genomes is difficult (Treangen and Salzberg 2011) and thus may result in underlying reference mistakes onto which the reads may map. However, research on human SVs has identified that centromeres and, more commonly, subtelomeric regions and telomeres are genomic regions where high proportions of SVs are observed (Collins et al. 2020), which (even accounting for the repeat filtering step) provides a biological explanation for these results. The SV density characterized by our short-read data was similar to that of avian corvid species, though SNP density was high in comparison to other species, which is possibly a result of different sampling schemes or filtering parameters choices (Weissensteiner et al. 2020).

We found that even in SVs up to lengths of 10 kb, the proportion of variants overlapping coding regions was similar to that of SNPs (each a single base pair in length). Characterization of standing structural variants in inbred lines of the plant species *Mimulus guttatus* (Flagel et al. 2014) found that SV indels overlapping with coding region regions were rarer than that would be expected by chance, with this high prevalence of noncoding and specifically repeat regions is seen in the profile of other avian species’ standing SVs (Weissensteiner et al. 2020; Peona et al. 2022). There is a growing appreciation for the potential functional role of the nonprotein coding regions that make up 98% of most eukaryotic genomes (Shihab et al. 2015), because variants within these regions can still impact an organism's phenotype (e.g., Zhang et al. 2018).

### Contrasting SNP and SV Patterns Across Populations of a Recent Invasive Species

Genetic structure assessed using PCA and Admixture analyses found similar results across the SNP and SV data for AU, UK, and NA individuals. AU individuals are genetically quite distinct from NA and UK across both SNP and SV datasets. This clustering pattern was previously identified in some of the individuals used as part of this study (Hofmeister et al. 2021), but importantly our dataset expanded the AU sampling scheme and therefore provides an important perspective on the extreme levels of genetic divergence at the western-most AU range-edge (Munglinup) using either SNPs or SVs. Previous studies using microsatellite, mitochondrial sequence, and reduced representation sequence data have identified high genetic differentiation at the western-most range-edge of AU starlings (Rollins et al. 2009, 2011; Stuart et al. 2021). This result is reflected too in our whole genome resequencing SNP and SV data. Considering the close proximity of Munglinup to the nearest other sampling site (Condingup), the genetic diversity loss as well as occurrence of unique genetic variants in both SNPs and SVs in Munglinup is interesting. Patterns of WGS and SV genetic variants identified at this range-edge may result from the undetected introduction(s) to Western Australia from a different (unsampled) part of the native range, novel selection within Western Australia, or both processes.

Heterozygosity measures across SNPs and SVs revealed a difference in genetic diversity patterns (H_O_, H_E_, *π*) across some sampling sites in our data, despite the above discussed the statistical correlation between SNP and SV densities. SNP data indicates a reduction in genetic diversity following introduction to Australia, whilst genetic diversity estimated from SVs is highest in AU starlings (table 1, supplementary table S3, Supplementary Material online). The relatively higher diversity of SVs (compared to patterns of SNP diversities between continents) in the AU range is not due to reference bias or batch effects, because this pattern remains even when the analysis was restricted to just a subset of individuals from the same sequencing batch, and when the reference genome, based on an AU individual, was replaced with a North American reference genome (*S. vulagirs* vNA; Stuart, Edwards et al. 2022). Follow-up analyses with a global pangenome (Chen et al. 2021) and long-read WGS data may help validate whether these results hold true for all types of SVs, or may reveal that these results only apply to the classes of SVs that are more easily detected by short-read SV callers, particularly DEL.

It seems apparent that at least for the SVs detected using our approach, AU individuals contain greater diversity than NA and UK individuals. If this pattern was due to sampling number of sample site distribution, we would expect these patterns to also be reflected in the SNPs, which was not seen in our data. Higher SV diversity in AU starlings could be a result of admixture of the multiple introductions into Australia. This hypothesis is supported by the fact that when genetic diversity was analyzed across all the AU samples [not just the Hofmeister et al. (2021) subset], it was Munglinup and Hobart that had the greatest relative increase in genetic diversity from SNPs and SVs. This is noteworthy because while admixture in Munglinup (discussed above) is only hypothesized, historical records corroborate mass admixture in Hobart as a result of approximately 100 starlings being imported from New Zealand in the late 1800s. It is also possible that the relatively high SV diversity in Australia is created or maintained through some demographic effects, as increased number of individuals during expansion will result in an increase in novel variants. Alternatively, it could be an adaptive response; it has been found in humans that there is an overrepresentation of SVs in genes that are important for interacting with the surrounding environment (e.g., olfaction and response to external stimuli; Feuk et al. 2006; Nguyen et al. 2006). The difference in SV and SNP allele frequency patterns have been found across multiple Asian rice varieties (Kou et al. 2020), thought to result at least in part from selection via domestication. The examination into how and why some populations have differing levels of genetic diversity across SNPs and SVs (and other forms of genetic variation) is integral for a better understanding of how genetic diversity is maintained within a population. As structural variant calling approaches improve and become more standardized across studies, investigation of broadscale patterns of relative SNP and SV densities across taxa will be useful. These studies can address questions about how mutational load may contribute to future population adaptation, despite the potential to confer deleterious effects on individuals (Wagner et al. 2017).

As would be expected of newly arisen variants, most of the AU-specific SV alleles were rare (fig. 6*a* and *b*), which contrasted strongly to the less variable site frequency spectrum (SFS) of the popgen-filtered SV dataset, and subsets. Within SVs with at least one break end overlapping a gene, we may expect an SFS preferencing lower MAF based on the potentially deleterious nature of the break end impact on coding region function, however, contrary to this we see higher MAFs are still relatively common (fig. 6*e*). It is possible that some of these SVs were small enough to be contained entirely within the same coding region and therefore have a non-negative (or even positive) effect on coding region function and hence selection, a supposition supported in part by the SFS of SVs containing entire genes showing even more frequent high MAFs (fig. 6*f*). Overall, the SFS plots indicate that global starling SVs with potentially functional effects are being maintained within the population, contrasting sharply to the much newer and rare AU-specific SVs. While most of the common AU-specific alleles were smaller than a few hundred base pairs in length, substantially longer ones were observed, and many overlapped with coding regions that may feasibly play a role in AU-specific starling evolution, for example, kidney function (ATP6V1B1; Müller et al. 2013) or immune system (FBXO16; Maertzdorf et al. 2011).

### Patterns of Selection in *Sturnus Vulgaris* SNPs and SVs

While environmental conditions may elicit directional selection on genetic variants, under fluctuating or intermediate environmental conditions, balancing selection may help to maintain genetic variation within populations, countering the tendency for polymorphisms to be lost via genetic drift. We found that the levels of native range balancing selection (calculated from SNPs) were significantly higher in nonoutlier SVs (DELs) compared to outlier and nonoutlier SNPs, suggesting balancing selection may play a greater role in maintain moderate SV allele frequencies compared to SNPs.

While we identified that balancing selection scores were higher in outlier SNPs than nonoutlier SNPs, we did not find the difference to be significant (supplementary table S5, Supplementary Material online). The results of the outlier SNP PCA (fig. 7*a*) compared to popgen-filtered SVs PCA (fig. 5*c*) support this trend because we see greater relative UK to AU spread in the former plot (though the same is not true for SVs; fig. 7*b*). In general, balancing selection scores were higher for SVs, with this trend becoming more pronounced using the alternate method for the pseudo *β*^(1)^ score calculation. However, it is likely that use of trimmed averages for each SV is the more appropriate approach because the trimmed average for each SNP helps to reduce overcontribution of linked SNPs with high *β*^(1)^ scores inflating the pseudo *β*^(1)^ scores for the SVs.

Despite previous assumptions that balancing selection may be a rare phenomenon (Fijarczyk and Babik 2015), there is a growing body of literature reporting widespread instances of this phenomenon facilitating adaptation under different selection regimes (de Filippo et al. 2016; Huang et al. 2016; Koenig et al. 2019). A recent study of the invasive copepod *Eurytemora affinis* found that genetic variants with signatures of long-term balancing selection in heterogenous native ranges were more likely to undergo parallel adaptive evolution in response to shifted environmental salinity levels within invasive ranges (Stern and Lee 2020). With this growing appreciation of the role of balancing selection in facilitating rapid adaption comes a need to better understand how selection regime shifts (e.g., land use change, climate change) reduce genetic diversity via loss of variants previously under balancing selection. Examining selection regime shifts relative to previous balancing selection equilibriums will be an important avenue of investigation to understand how declining native populations, such as in the starling (Heldbjerg et al. 2016; Versluijs et al. 2016), may spiral further toward detrimental genetic diversity loss and collapse. Our results support the hypothesis that balancing selection plays a key role in maintain genetic variants and indicates that different types of genetic variants may experience different levels of balancing selection within a population.

### Challenges in SV Calling and Genotyping

Our study is one of the first to look at patterns of population genetics and selection across genome-wide SNPs and SVs. In doing so, we highlight some important pitfalls in SV data analysis using whole genome resequenced population genetic data. In many WGS studies attempting to characterize SVs, resequencing costs prohibit large sample sizes, and larger sample sizes may be required to fully investigate genomic responses to environmental change. We acknowledge that our study of the native range would benefit from a larger sample size. It is common for studies employing WGS data to combine newly sequenced individuals with WGS data from previous studies because higher sequencing costs and DNA quality requirements necessitate sample reuse. However, as evidenced by the results of this study, while batch effects are often not a major concern in WGS SNP calling (due to higher certainty), we found that sequencing batch effects impacted our SV data—specifically heterozygosity increased with sequencing depth (supplementary fig. S1, Supplementary Material online). While combining WGS data cannot and should not be avoided where it can expand sample coverage and study scope, caution should be used and batch effects explicitly tested so that downstream analyses may be validated with this in mind. This can be handled by restricting genetic diversity comparisons to just a single sequencing batch of individuals, as we have done here.

Despite these SV calling and genotyping uncertainties, population genetics analysis using SNP and SV datasets, in particular Admixture, captured very similar patterns of overall differentiation across the individuals included in this study. The finding is reassuring because previous studies have demonstrated that SV genotyping may have many false positives and negatives (Chakraborty et al. 2019; Weissensteiner et al. 2020). Technical difficulties, such as when the variant spans the sequencing molecule's length many times over, the repeat content within the variant, or the overall complexity of the rearrangement, means that short-read sequencing is particularly prone to SV genotyping errors (Chander et al. 2019; Ho et al. 2020; Wold et al. 2021). Hence it is reassuring to find that broad patterns of genetic variation were quite similar across the high-confidence SNP and lower-confidence SV dataset. We note here that the congruence between population genetic patterns between the SNP and SV dataset was greatly improved from earlier dataset versions through modification of the Survivor pipeline. The inclusion of genotype information as criteria for merging SV calls and retaining genotype information for each sample improved sample clustering, and thus we suggest this may be an improvement on the usage of this program for those wishing to use SV data in population genetics studies.

We note here that the SV diversities obtained from the *S. vulgaris* vNA reference genome were much higher, in contrast to the SV diversity levels obtained from the *S. vulgaris* vAU1.0 genome (which were lower also relative to SNPs). We do not focus on the comparison between these results, because the vNA genome was much less contiguous, which likely contributed to the increased SV calls and higher heterozygosity. Instead, we emphasize the relative trends between sample groups within each dataset rather than absolute differences. This result does, however, demonstrate just how variable SV datasets generated from different genome versions may be, particularly in comparison to SNPs that are far more consistent (e.g., for WGS SNP comparison across three genome versions see Hofmeister et al. (2021) appendix).

## Conclusion

The findings of our research demonstrate that even within recently diverged lineages or populations, there may be high amounts of SV variation. Further, patterns of SV genetic diversity do not necessarily reflect relative patterns in SNP diversity, either when considering patterns of diversity along the length of the organism's chromosomes (owing to enrichment of SVs in subtelomeric repeat regions), or interpopulation diversity patterns (possibly a result of altered selection regimes or introduction history). Finally, we found that levels of balancing selection within the native range differed across SNP and SV groupings and were higher in SVs, suggesting that selection regimes may favor different optimum frequencies for different types of genetic variants. Overall, our results demonstrate that the processes that shape allelic diversity within populations are complex and illustrate the need for further investigation of SVs across a range of taxa to better understand correlations between oft well-studied SNP diversity and that of SVs.

## Supplementary Material

msad046_Supplementary_Data

## Data Availability

Whole genome resequencing read data for individuals au09-au33 are available under the SRA accession PRJNA935977. Whole genome resequencing read data for individuals au01-08, us01-us08, and uk01-08 are available via the published Hofmesiter et al. (2021) manuscript through the SRA accession PRJNA938765. Code is available at https://github.com/katarinastuart/Sv5_StarlingWGS.
