# Supplementary material for: Contrasting Patterns of Single Nucleotide Polymorphisms and Structural Variation Across Multiple Invasions
Source: Mol Biol Evol. 2023 Feb 23;40(3):msad046. doi: 10.1093/molbev/msad046 (PMC10037079; doi:10.1093/molbev/msad046)
Supplement: msad046_Supplementary_Data [file msad046_supplementary_data.pdf]

Contrasting patterns of single nucleotide polymorphisms and structural variations across multiple invasions

Katarina C. Stuart[1,2], Richard J. Edwards[3], William B. Sherwin[1], Lee A. Rollins[1]

[1] Evolution & Ecology Research Centre, School of Biological, Earth and Environmental Sciences, UNSW Sydney, Sydney, New South Wales, Australia

[2] School of Biological Sciences, University of Auckland, Auckland, New Zealand

[3] Evolution & Ecology Research Centre, School of Biotechnology and Biomolecular Sciences, UNSW Sydney, Sydney, New South Wales, Australia

Supplementary Materials

Appendix 1: Whole genome resequencing of the *Sturnus vulgaris* whole genomes

Methods

Individuals au01-au33 were collected from South Australia and Western Australia from 2003 – 2007 (Rollins et al. 2009), and from the south-eastern portion of the Australian range from 2011-2012 (Stuart et al. 2021). In both sets of samples, birds were collected in three ways: i) by trapping, ii) as carcasses collected from 3rd party hunters or landowners that humanely killed birds on their property, or iii) chicks collected directly from nests. In the latter case, only one individual per family (adult or chick) was included in the dataset to avoid the inclusion of closely related individuals. Muscle was sampled from the thigh or breast, or liver was taken from a dissected bird, and stored it in 70% ethanol or snap frozen in liquid nitrogen, and stored long-term in -80°C freezers. Individuals au01-au08, us 01-us08, and uk01-uk08 were included as part of an earlier study (Hofmeister et al. 2021). DNA extractions were performed using the QIAGEN Gentra Puregene Tissue kit as per manufacturer's instructions. For individuals au01-au14, us01-us08, and uk01-uk08, libraries for each individual starling were constructed using a TruSeq DNA PCR-free Library Prep Kit (Illumina, San Diego, CA) and sequenced on a HiSeqX at the McDonnell Genome Institute (Missouri, USA). For individuals au15-au30, libraries for each individual starling were constructed using a TruSeq DNA PCR-free Library Prep Kit and sequenced on a NovaSeq6000 at Deakin University Core Sequencing facility (Geelong, Australia). For individuals au31-au33, libraries for each individual starling were constructed using a TruSeq DNA PCR-free Library Prep Kit and sequenced on a NovaSeq6000 at Australian Genome Research Facility (Melbourne, Australia).

Results

Short read whole genome resequencing of the 49 individual starlings yielded total read counts from 103,209,440 - 630,512,935 per individual (Table S1).

Table S1: Summary of WGS *Sturnus vulgaris* individuals used for whole genome SNP and SV calling, along with batch, read count and read depth information. Sample mean depth per individual calculated by VCFTOOLS on the thinned SNP dataset.

IND.	Pop. Status	Country	Region	Location	AU subcluster	Batch	Total Read Count	Read Depth (SNP)
au01	Invasive	Australia	NSW	Lemon Tree	East	1	161,774,866	16.4
au02	Invasive	Australia	NSW	Lemon Tree	East	1	138,481,596	14.1
au03	Invasive	Australia	NSW	Maitland	East	1	121,949,997	12.0
au04	Invasive	Australia	NSW	Maitland	East	1	121,934,492	12.6
au05	Invasive	Australia	SA	Meningie	South	1	121,963,487	12.6
au06	Invasive	Australia	SA	Meningie	South	1	130,652,648	13.5
au07	Invasive	Australia	VIC	Wonthaggi	South	1	130,147,119	14.5
au08	Invasive	Australia	VIC	Wonthaggi	South	1	159,551,759	15.9
au09	Invasive	Australia	WA	Munglinup	South	1	168,989,017	16.7
au10	Invasive	Australia	NSW	Hay	East/South	1	118,574,348	12.3
au11	Invasive	Australia	WA	Munglinup	South	1	120,434,420	12.8
au12	Invasive	Australia	WA	Condingup	South	1	115,005,268	12.2

au13	Invasive	Australia	NSW	Hay	East/South	1	132,738,406	13.5
au14	Invasive	Australia	NSW	Dubbo	East	1	110,957,661	11.3
au15	Invasive	Australia	SA	Meningie	South	2	128,379,920	13.7
au16	Invasive	Australia	WA	Munglinup	South	2	260,596,607	26.4
au17	Invasive	Australia	WA	Munglinup	South	2	224,492,296	22.7
au18	Invasive	Australia	WA	Condingup	South	2	211,097,422	21.6
au19	Invasive	Australia	WA	Condingup	South	2	218,651,503	22.5
au20	Invasive	Australia	TAS	Hobart	South	2	215,058,859	22.5
au21	Invasive	Australia	TAS	Hobart	South	2	329,774,102	32.0
au22	Invasive	Australia	TAS	Hobart	South	2	266,604,570	26.7
au23	Invasive	Australia	NSW	Dubbo	East	2	198,731,521	20.2
au24	Invasive	Australia	NSW	Dubbo	East	2	230,443,227	24.0
au25	Invasive	Australia	NSW	Hay	East/South	2	211,675,237	21.6
au26	Invasive	Australia	NSW	Lemon Tree	East	2	243,645,414	24.7
au27	Invasive	Australia	NSW	Maitland	East	2	259,735,087	26.6
au28	Invasive	Australia	WA	Munglinup	South	2	239,006,061	24.3
au29	Invasive	Australia	WA	Munglinup	South	2	264,975,415	26.6
au30	Invasive	Australia	VIC	Wonthaggi	South	2	235,789,158	22.6
au31	Invasive	Australia	WA	Munglinup	South	3	390,701,211	37.5
au32	Invasive	Australia	WA	Munglinup	South	3	630,512,935	59.0
au33	Invasive	Australia	WA	Munglinup	South	3	503,636,307	47.7
us01	Invasive	USA	NY	New York	-	1	170,518,757	12.4
us02	Invasive	USA	NY	New York	-	1	127,905,364	17.1
us03	Invasive	USA	NY	New York	-	1	104,086,567	16.5
us04	Invasive	USA	NY	New York	-	1	126,123,487	14.7
us05	Invasive	USA	NY	New York	-	1	103,209,440	13.8
us06	Invasive	USA	NY	New York	-	1	140,354,835	14.7
us07	Invasive	USA	NY	New York	-	1	150,480,727	15.8
us08	Invasive	USA	NY	New York	-	1	132,168,818	15.4
uk01	Native	England	UK	Newcastle upon Tyne	-	1	122,402,512	18.3
uk02	Native	England	UK	Newcastle upon Tyne	-	1	166,651,716	13.7
uk03	Native	England	UK	Newcastle upon Tyne	-	1	160,423,348	10.9
uk04	Native	England	UK	Newcastle upon Tyne	-	1	143,343,371	13.3
uk05	Native	England	UK	Newcastle upon Tyne	-	1	134,177,188	11.3
uk06	Native	England	UK	Newcastle upon Tyne	-	1	138,343,599	15.0
uk07	Native	England	UK	Newcastle upon Tyne	-	1	154,792,665	16.2
uk08	Native	England	UK	Newcastle upon Tyne	-	1	150,807,756	14.2

Table S2: Summary of structural variant (SV) calls across all *Sturnus vulgaris* individuals across three different SV callers LumpySV, Delly, and Manta. DEL = deletion, DUP = duplication, INS = insertion, INV = inversion, TRA = Breakend

	DEL	DUP	INS	INV	TRA
**Lumpy SV**	42243	13649	0	698	138426
**Delly**	43813	2054	8169	3387	12366
**Manta**	27994	543	27064	1172	1328

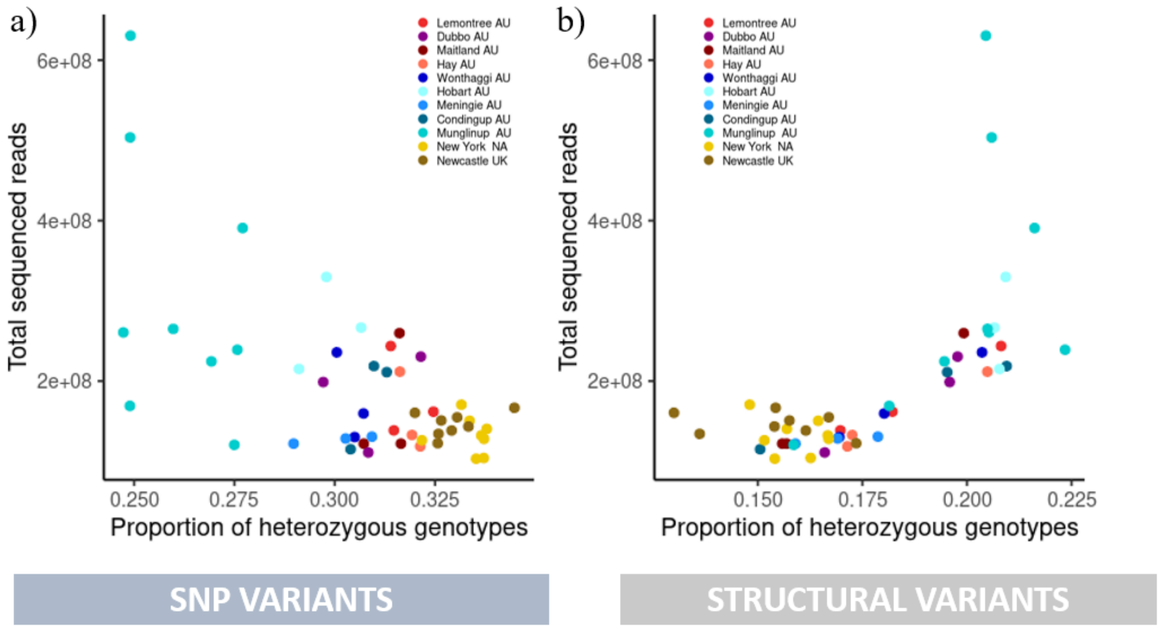

Figure S1: Total sequenced read count verses heterozygous genotype proportion across *Sturnus vulgaris* samples for SNPs and SV variants, coloured by sample site. No effect of read counts on individual heterozygosity rates using a linear mixed model, with population set as a random factor for SNP variants (β=-0.104, SE= 0.0613, z(49)= -1.692). A significant effect was found for read counts on individual heterozygosity rates using a linear mixed model, with just population set as a random factor (β=0.5205, SE=0.111, z(49)= 4.692, p-value < 0.001), which was no longer present when sequencing batch was also added as a random factor to the model (β=0.2691, SE=0.158, z(49)= 1.709).

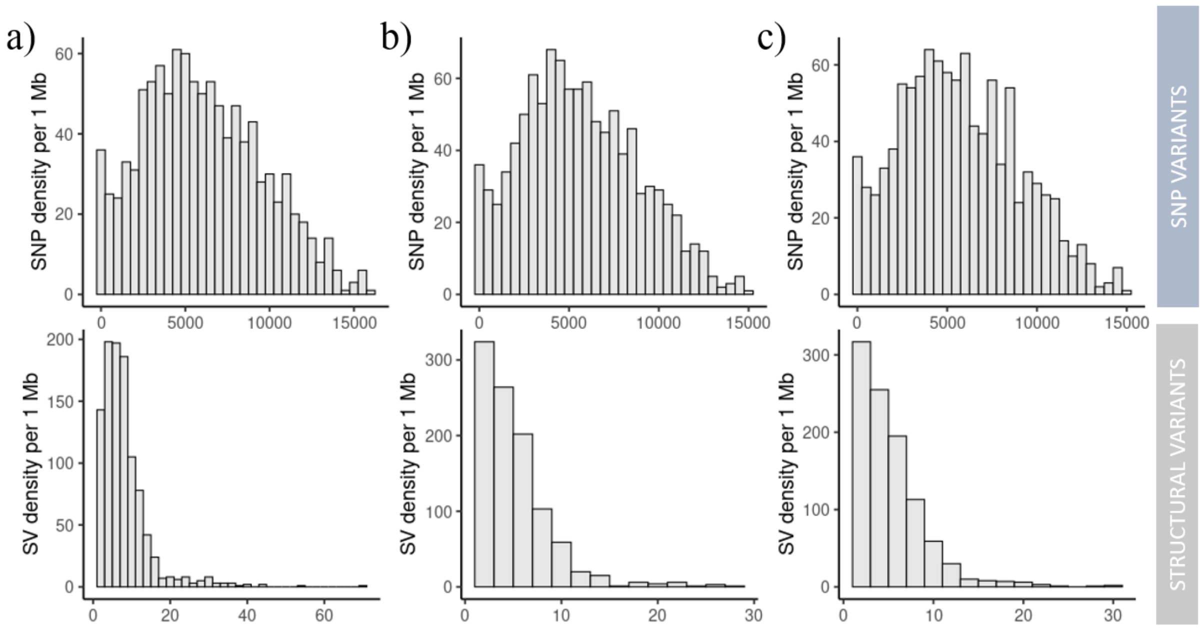

Figure S2: Density histograms (1 Mb windows) of SNP and SV in *Sturnus vulgaris* across the sampled continents, with panel a) invasive AU, panel b) invasive NA, and panel c) native range UK samples.

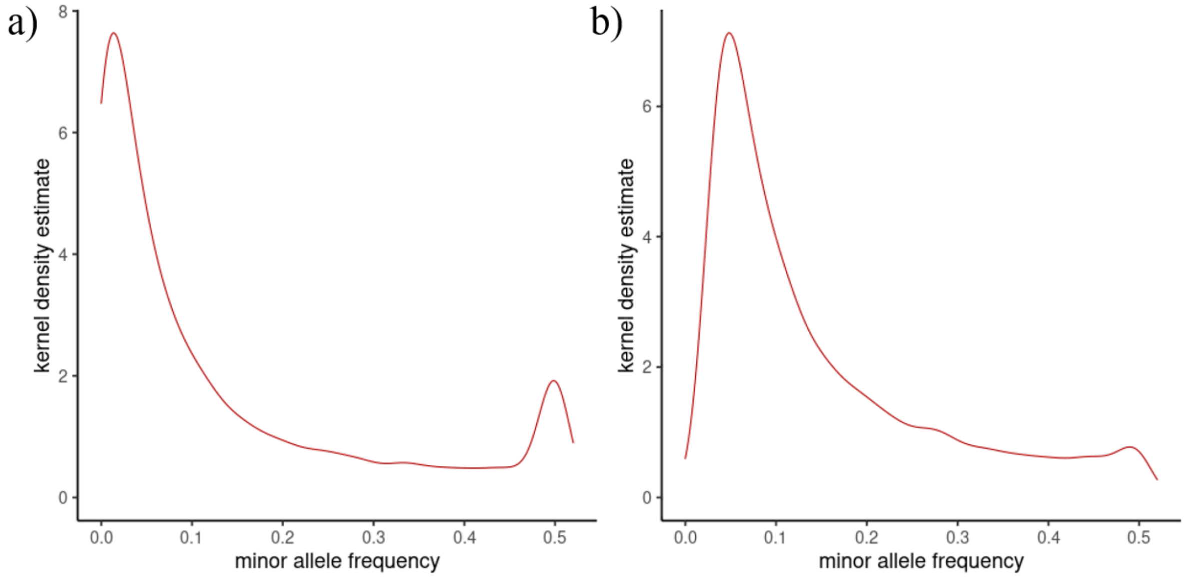

Figure S3: Minor allele frequency kernel density estimated for *Sturnus vulgaris* **structural variants**, with panel a) unfiltered SV dataset, and panel b) the popgen filtered SV dataset .

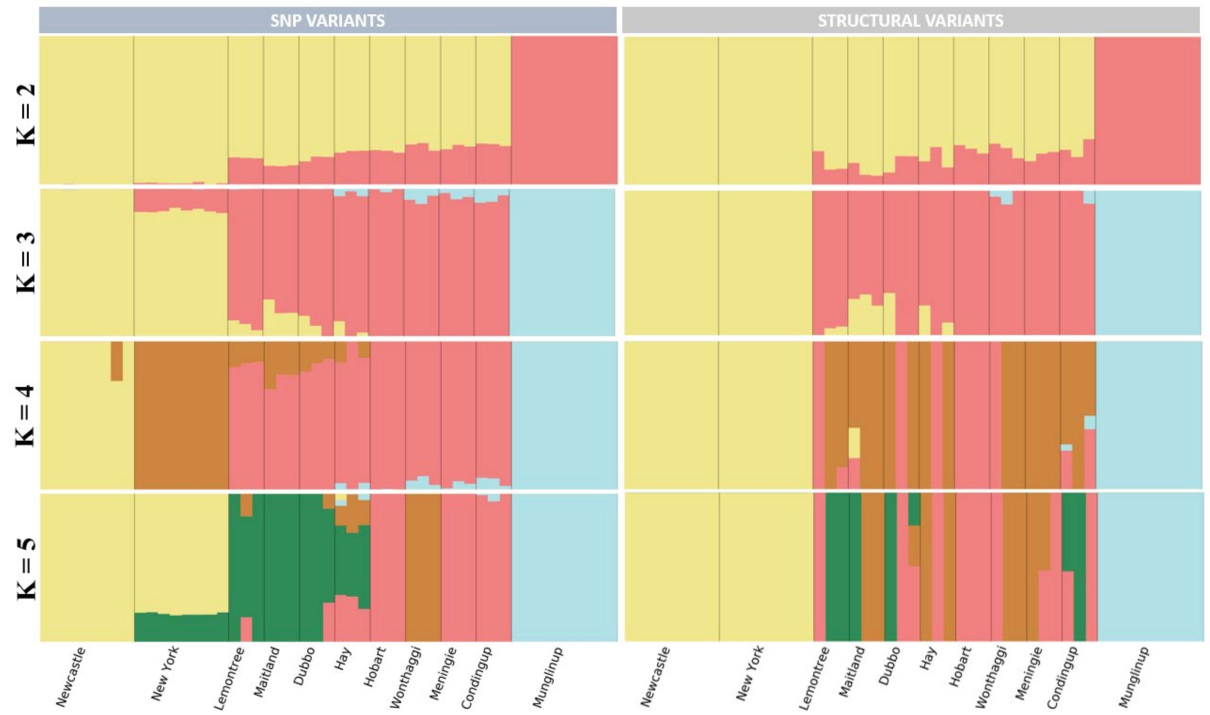

Figure S4: Admixture plots SNP and SV in *Sturnus vulgaris* across K values 2-5.

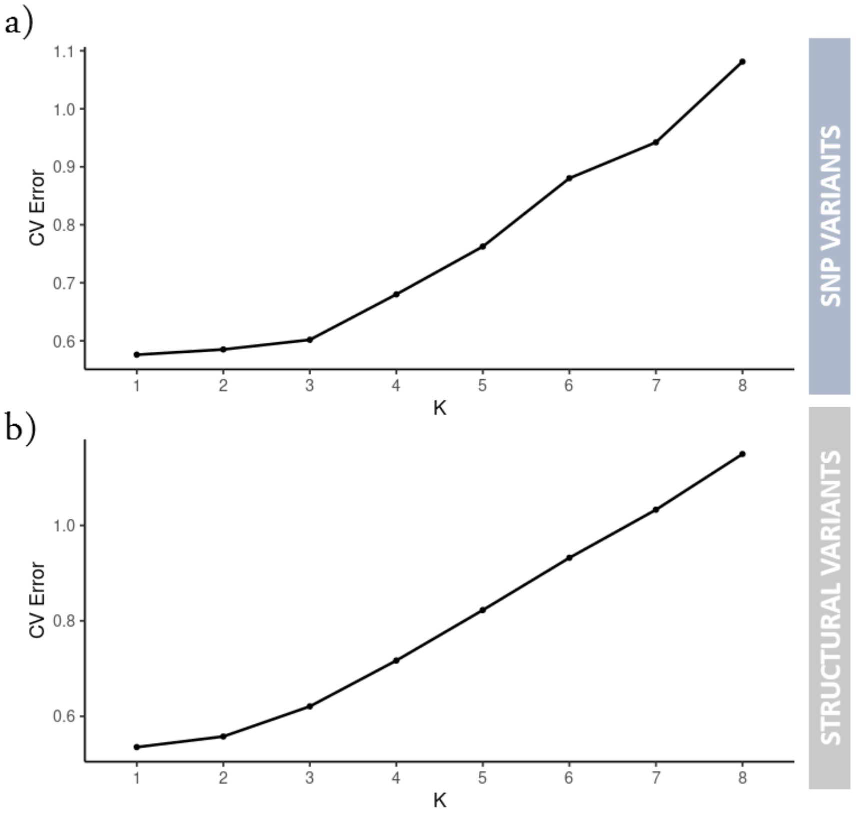

Figure S5: Admixture cross-validation error plots SNP and SV in Sturnus vulgaris across K values 1-8.

Table S3: Single nucleotide polymorphism (SNP) and strucutral variant (SV) summary statistics for *Sturnus vulgaris* across study sample sites and continents. H_O, H_E, and $\boldsymbol{\pi}$ values represent averages for each sampling group, with the standard error in brackets.

Continent	Sampling Group (sample number)		Single nucleotide polymorphisms (SNP)			
			Thinned SNP dataset (186,205)			
			No. Polymorphic Sites	H_O (StdErr)	H_E (StdErr)	π (StdErr)
**Australia** *Invasive*	All sites (33)		167,868	0.2716 (0.0004)	0.2777 (0.0004)	0.282 (0.0004)
		Lemon Tree (3)	111,778	0.2935 (0.0007)	0.2416 (0.0005)	0.29 (0.0006)
		Maitland (3)	111,813	0.2908 (0.0007)	0.2415 (0.0005)	0.29 (0.0006)
		Dubbo (3)	111,928	0.2853 (0.0007)	0.2417 (0.0005)	0.2901 (0.0006)
		Hay (3)	112,122	0.2948 (0.0007)	0.2424 (0.0005)	0.291 (0.0006)
		Wonthaggi (3)	107,553	0.2797 (0.0007)	0.2338 (0.0005)	0.2806 (0.0006)
		Hobart (3)	105,256	0.2736 (0.0007)	0.2276 (0.0005)	0.2732 (0.0006)
		Meningie (3)	107,729	0.2775 (0.0007)	0.2334 (0.0005)	0.2802 (0.0006)
		Condingup (3)	107,024	0.2838 (0.0007)	0.2322 (0.0005)	0.2788 (0.0006)
		Munglinup (9)	111,933	0.2363 (0.0006)	0.2122 (0.0005)	0.2247 (0.0005)
	Hofmeister et al. Subset (8)		146,766	0.2857 (0.0005)	0.2723 (0.0004)	0.2905 (0.0004)
		$Australia_{EAST}$ (4)	124,050	0.2926 (0.0006)	0.2541 (0.0005)	0.2905 (0.0005)
		$Australia_{SOUTH}$ (4)	120,450	0.2788 (0.0006)	0.2483 (0.0005)	0.2839 (0.0005)
**North America** *Invasive*		New York (8)	154,991	0.3108 (0.0005)	0.2888 (0.0004)	0.3081 (0.0004)
**United Kingdom** *Native*		Newcastle upon Tyne (8)	158,574	0.3152 (0.0005)	0.2946 (0.0004)	0.3142 (0.0004)

H_O = Observed Heterozygosity, H_E = the within population gene diversity, F_{IS} = inbreeding coefficient, No. polymorphic site indicates the number of nucleotide positions that are polymorphic in at least one indivdual within a sample site or sample grouping. Number of polymorphic sites, H_O, H_E, and F_{IS} were assessed using STACKS *populations*. Variance and standard error information available in Table S3. SNP dataset was the thinned SNP dataset (186,205 SNPs), and SV datasets were the popgen-filtered SV dataset (9,110 SVs), and an alternate version of this that had no minor allele frequency (MAF) filtering (7,898 SVs).

Table S3 (CONT): Single nucleotide polymorphism (SNP) and strucutral variant (SV) summary statistics for *Sturnus vulgaris* across study sample sites and continents. H_O, H_E, and F_{IS} values represent averages with the standard error in brackets.

Continent	Sampling Group (sample number)		Structural Variants (SV)							
			S. vulgaris vAU genome (9,110)				*S. vulgaris* vNA genome (22,616)			
			No. Polymorphic Sites	H_O (StdErr)	H_E (StdErr)	π (StdErr)	No. Poly-morphic Sites	H_O (StdErr)	H_E (StdErr)	π (StdErr)
**Australia** *Invasive*	All sites (33)		8,793	0.1916 (0.0019)	0.2314 (0.0016)	0.2355 (0.0016)	-	-	-	-
		Lemon Tree (3)	4,065	0.189 (0.003)	0.1693 (0.0021)	0.2115 (0.0027)	-	-	-	-
		Maitland (3)	3,864	0.1747 (0.0029)	0.1621 (0.0021)	0.2033 (0.0027)	-	-	-	-
		Dubbo (3)	4,201	0.1897 (0.0029)	0.1753 (0.0021)	0.2188 (0.0027)	-	-	-	-
		Hay (3)	4,035	0.1854 (0.0029)	0.1674 (0.0021)	0.2093 (0.0027)	-	-	-	-
		Wonthaggi (3)	4,107	0.1878 (0.003)	0.1728 (0.0021)	0.2162 (0.0027)	-	-	-	-
		Hobart (3)	4,267	0.211 (0.0031)	0.1805 (0.0021)	0.2254 (0.0027)	-	-	-	-
		Meningie (3)	3,874	0.1721 (0.0029)	0.1632 (0.0021)	0.2044 (0.0027)	-	-	-	-
		Condingup (3)	4,001	0.1884 (0.003)	0.169 (0.0021)	0.212 (0.0027)	-	-	-	-
		Munglinup (9)	5,699	0.205 (0.0026)	0.2037 (0.002)	0.2194 (0.0022)	-	-	-	-
	Hofmeister et al. Subset (8)		6,281	0.1722 (0.0022)	0.1966 (0.0018)	0.2128 (0.002)	19,445	0.4823 (0.0025)	0.3269 (0.0012)	0.3559 (0.0013)
		$Australia_{EAST}$ (4)	4,447	0.1696 (0.0026)	0.1695 (0.002)	0.2003 (0.0024)	16,770	0.4815 (0.0027)	0.3092 (0.0014)	0.3748 (0.0017)
		$Australia_{SOUTH}$ (4)	4,594	0.1746 (0.0026)	0.1787 (0.002)	0.2104 (0.0024)	16,819	0.4813 (0.0027)	0.3112 (0.0014)	0.3766 (0.0017)
**North America** *Invasive*		New York (8)	5,451	0.1586 (0.0023)	0.173 (0.0019)	0.1874 (0.002)	19,631	0.4793 (0.0025)	0.323 (0.0012)	0.352 (0.0013)
**United Kingdom** *Native*		Newcastle upon Tyne (8)	5,593	0.1627 (0.0023)	0.1746 (0.0018)	0.1891 (0.002)	19,843	0.4859 (0.0025)	0.3267 (0.0012)	0.3555 (0.0013)

H_O = Observed Heterozygosity, H_E = the within population gene diversity, F_{IS} = inbreeding coefficient, No. polymorphic site indicates the number of nucleotide positions that are polymorphic in at least one indivdual within a sample site or sample grouping. Number of polymorphic sites, H_O, H_E, and F_{IS} were assessed using STACKS *populations*. Variance and standard error information available in Table S3. SNP dataset was the thinned SNP dataset (186,205 SNPs), and SV datasets were the popgen-filtered SV dataset (9,110 SVs), and an alternate version of this that had no minor allele frequency (MAF) filtering (7,898 SVs).

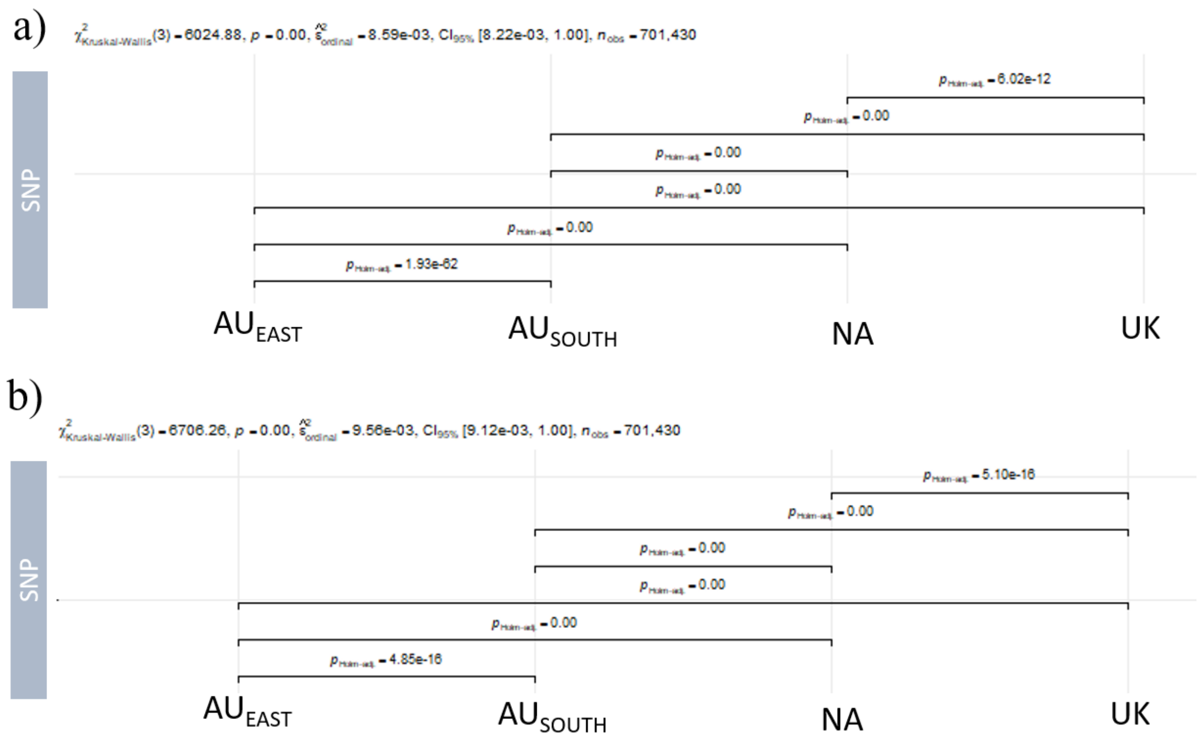

Figure S6: Nonparametric between group comparison of genetic diversity measures across sample sites for popgen filtered SNPs (186,205) in *Sturnus vulgaris*, with panel a) depicting H_O (observed heterozygosity) and panel b) H_E (expected heterozygosity). Pairwise testing via Dunn test, with only significant pairwise differences displayed.

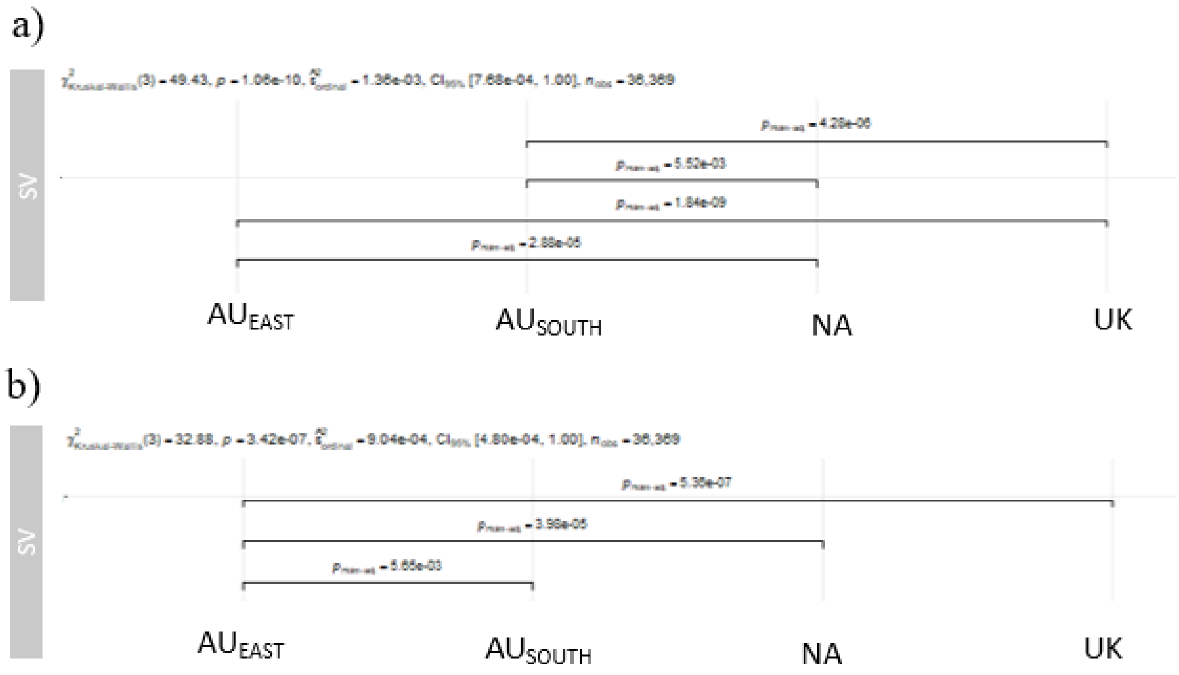

Figure S7: Nonparametric between group comparison of genetic diversity measures across sample sites for popgen filtered structural variants (9,110) in *Sturnus vulgaris*, with panel a) depicting H_O (observed heterozygosity) and panel b) H_E (expected heterozygosity). Pairwise testing via Dunn test, with only significant pairwise differences displayed.

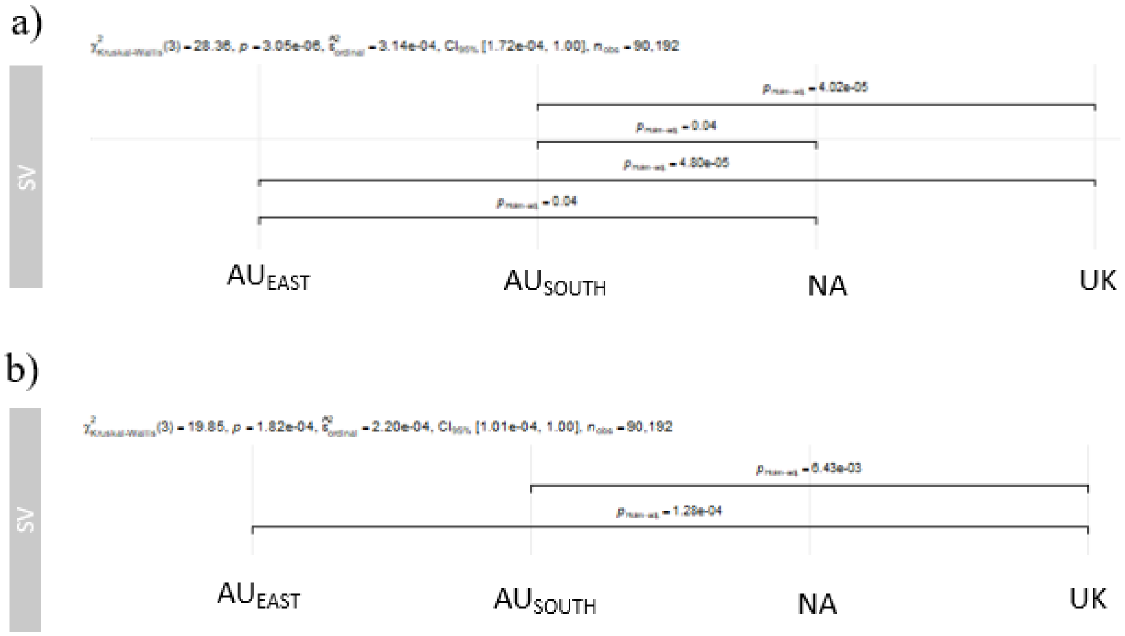

Figure S8: Nonparametric between group comparison of genetic diversity measures across sample sites for vNA popgen filtered structural variants (22,616) in *Sturnus vulgaris*, with panel a) depicting H_O (observed heterozygosity) and panel b) H_E (expected heterozygosity). Pairwise testing via Dunn test, with only significant pairwise differences displayed.

Table S4: List of 1 kb plus common private SV alleles within Australia, and the genes that they overlap with or exist within 1 kb of the ends of the structural variant.

CHROM	POS	TYPE	LENGTH	GENES
chromosome3	204927	DUP	316614	FBXO16: F-box only protein 16<br>Fzd3: Frizzled-3<br>EXTL3: Exostosin-like 3
chromosome3	19660693	DEL	7945	env: Envelope glycoprotein gp95
chromosome3	19431228	DEL	6930	ERVK-8: Endogenous retrovirus group K member 8 Gag polyprotein<br>ERVK-11: Endogenous retrovirus group K member 11 Pol protein
chromosomeZ	53804790	DEL	5028	
chromosome3	57291865	DEL	1647	
chromosome3	54343523	DEL	1572	NKAIN2: Sodium/potassium-transporting ATPase subunit beta-1-interacting protein 2
chromosome4	1277249	DEL	702	EXOC6B: Exocyst complex component 6B
chromosome4	44243248	DEL	665	
chromosome23	3146470	DEL	558	MIMI_R196: Collagen-like protein 2<br>ADGRB2: Adhesion G protein-coupled receptor B2<br>EVI5L: EVI5-like protein<br>PELPK1: Protein PELPK1<br>fhaB: Filamentous hemagglutinin
chromosome5	23632309	DEL	508	
chromosome27	1815034	DEL	485	ARHGAP23: Rho GTPase-activating protein 23
chromosome12	2711790	DEL	404	NT5DC2: 5'-nucleotidase domain-containing protein 2<br>Smim4: Small integral membrane protein 4
chromosome2	14007651	DEL	400	ACTR3B: Actin-related protein 3B
chromosomeZ	49124371	DEL	378	
chromosome3	47323072	DEL	364	Sec63: Translocation protein SEC63 homolog
chromosomeZ	49143571	DEL	239	
chromosome9	5622558	DEL	235	Kcnab1: Voltage-gated potassium channel subunit beta-1
chromosome4	6449901	DEL	233	Nagk: N-acetyl-D-glucosamine kinase<br>ATP6V1B1: V-type proton ATPase subunit B kidney isoform
chromosome4	41221659	DEL	232	
chromosome4	63705519	DEL	223	

chromosome3	85981659	DEL	196	Rps6ka2: Ribosomal protein S6 kinase alpha-2
chromosome21	7937217	DEL	192	MINOS1: MICOS complex subunit MIC10
chromosome21	1534764	DEL	191	
chromosome4	6563514	DEL	184	COL9A3: Collagen alpha-3(IX) chain<br>vax2-a: Ventral anterior homeobox 2a<br>coe3: Transcription factor COE3
chromosome6	16913081	DEL	182	ANXA7: Annexin A7<br>EIF3F: Eukaryotic translation initiation factor 3 subunit F
chromosome7	37684722	DEL	182	Nebl: LIM zinc-binding domain-containing Nebulette<br>RIF1: Telomere-associated protein RIF1
chromosome1A	71410491	DEL	181	
chromosome5	47932314	DEL	173	
chromosome4A	2271643	DEL	172	CHML: Rab proteins geranylgeranyltransferase component A 2
chromosome19	7438574	DEL	171	
chromosomeZ	3500317	DEL	167	Rit2: GTP-binding protein Rit2
chromosome26	4667173	DEL	167	
chromosome22	2409882	DEL	161	
chromosome2	88159083	DEL	160	
chromosome21	821240	DEL	160	
chromosome28	3395323	DEL	155	PTPRS: Receptor-type tyrosine-protein phosphatase S
chromosome5	37359115	DEL	154	
chromosome24	4297343	DEL	151	

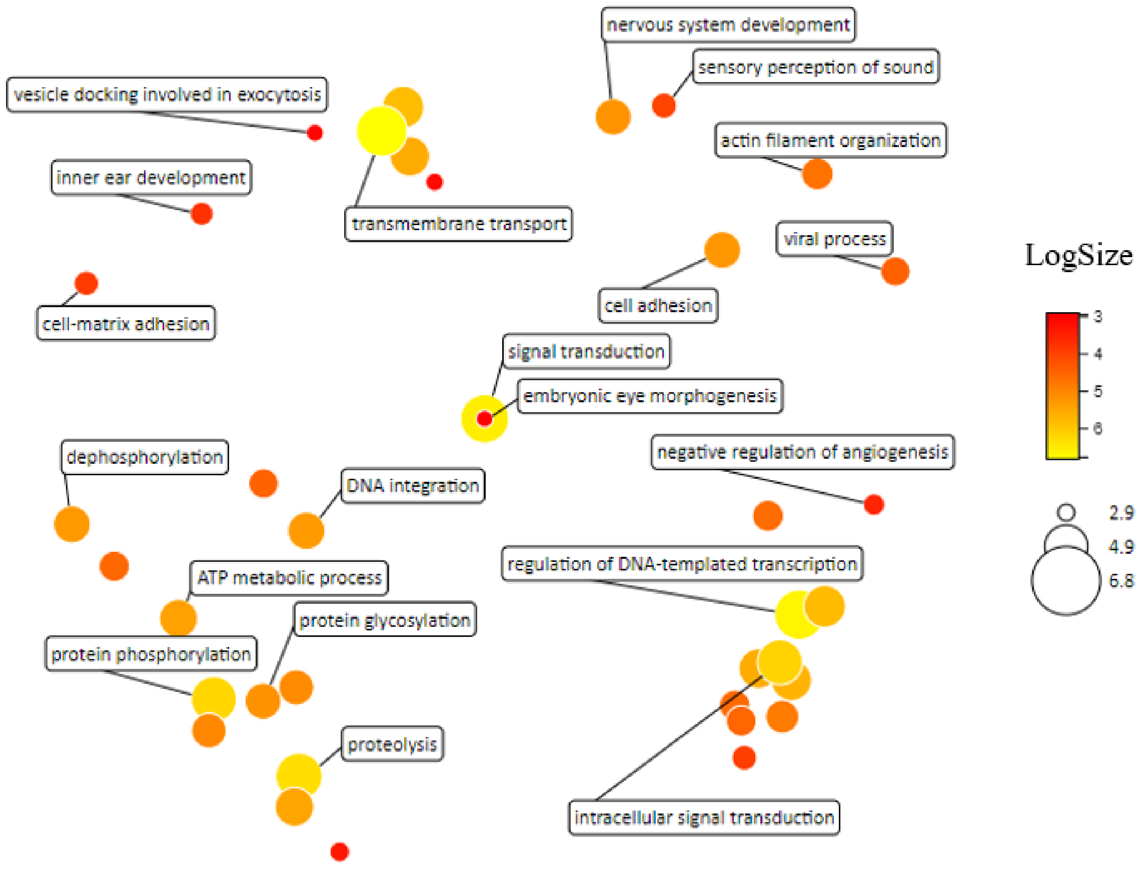

Figure S9: Summary of gene ontology (GO) biological processes for common unique SVs in *Sturnus vulgaris*, summarised using REVIGO. The scatterplot shows representative clusters of the GO analysis, with Log Size (indicated by circle diameter and colour) representing the frequency of GO term in the dataset, plotted in semantic space with similar GO terms being placed close to one another.

Table S5: Dunn's test of multiple comparisons of pairwise balancing selection differences between sample groups for SNPs and SVs under directional selection (outliers) and not under directional selection (nonoutliers) between AU and UK, calculated in R using the *dunnTest()* function as a follow up to a significant *kruskal.test()* result (Kruskal-Wallis chi-squared = 647.88, df = 6, p-value < 2.2e-16). (Significant level indicated by: 0.05 > p-value ≥ 0.01 = *, 0.01 > p-value ≥ 0.001 = **, 0.001 > p-value = ***). INS removed from analysis due to a sample size of 1.

**Comparison**	**Z Test Statistic**	**Adjusted p-values**	**Sig.**
SNPoutlier-SNPnonoutlier	0.401644	1.00E+00	
SNPoutlier-SVoutlier	-2.46522	2.05E-01	
SNPoutlier-SVnonoutliersDEL	-6.182	1.27E-08	***
SNPoutlier-SVnonoutliersDUP	-2.01835	4.36E-01	
SNPoutlier-SVnonoutliersTRA	-1.45349	1.00E+00	
SNPoutlier-SVnonoutliersINV	2.081003	4.49E-01	
SNPnonoutlier-SVoutlier	-2.92807	6.48E-02	
SNPnonoutlier-SVnonoutliersDEL	-25.0854	1.51E-137	***
SNPnonoutlier-SVnonoutliersDUP	-2.27135	3.01E-01	
SNPnonoutlier-SVnonoutliersTRA	-1.91396	4.45E-01	
SNPnonoutlier-SVnonoutliersINV	2.056639	4.37E-01	
SVoutlier-SVnonoutliersDEL	-0.33679	1.00E+00	
SVoutlier-SVnonoutliersDUP	-0.14956	1.00E+00	
SVoutlier-SVnonoutliersTRA	1.035571	1.00E+00	
SVoutlier-SVnonoutliersINV	2.482548	2.09E-01	
SVnonoutliersDEL-SVnonoutliersDUP	0.058035	9.54E-01	
SVnonoutliersDEL-SVnonoutliersTRA	2.011264	3.99E-01	
SVnonoutliersDEL-SVnonoutliersINV	2.564707	1.86E-01	
SVnonoutliersDUP-SVnonoutliersTRA	0.983457	1.00E+00	
SVnonoutliersDUP-SVnonoutliersINV	2.493794	2.15E-01	
SVnonoutliersTRA-SVnonoutliersINV	2.284452	3.13E-01	

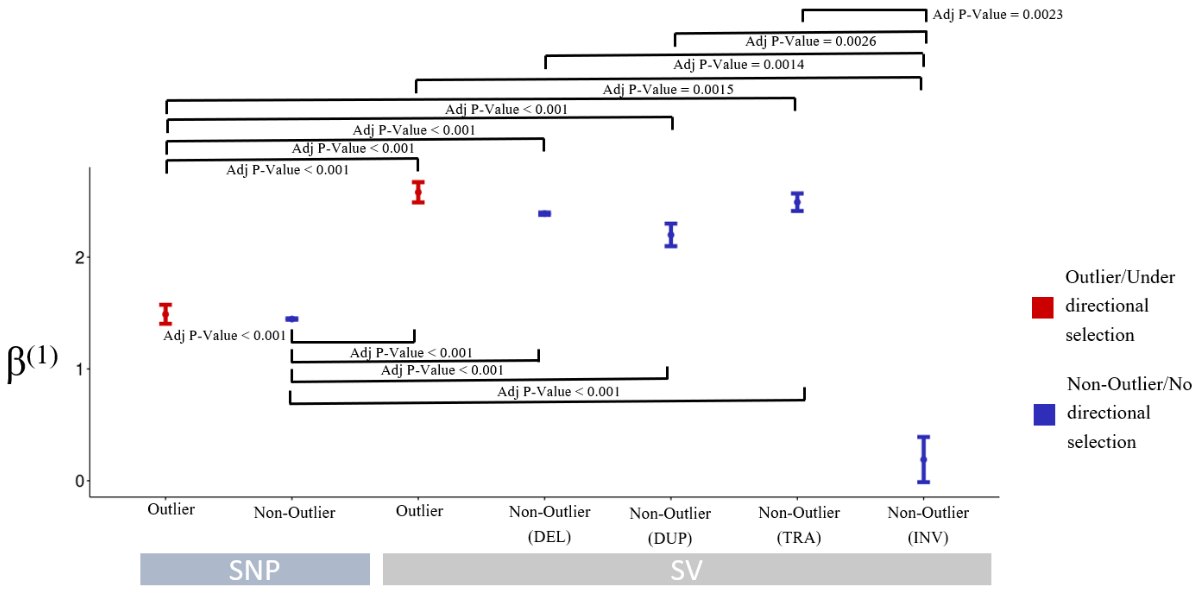

Figure S10: Genetic variants under putative directional selection in *Sturnus vulgaris* across the native range and invasive Australian range and assessment of balancing selection, with mean $\beta^{(1)}$ scores (balancing selection within the native UK range) for SNPs and SVs under directional selection (BAYESCAN and BAYPASS outliers between AU and UK) and no directional selection (+/- standard error) in UK individuals, alongside the adjusted p-value of Dunn's test as a follow up to a significant *kruskal.test()* result (Kruskal-Wallis chi-squared = 7325.1, df = 6, p-value < 2.2e-16). SV pseudo $\beta^{(1)}$ scores calculated by averaging all overlapped SNP $\beta^{(1)}$ scores within each SV type group. INS removed from analysis due to a sample size of 1.

Table S6: Dunn's test of multiple comparisons of pairwise balancing selection differences between sample groups for SNPs and SVs under directional selection (outliers) and not under directional selection (nonoutliers) between AU and UK, calculated in R using the *dunnTest()* function as a follow up to a significant *kruskal.test()* result (Kruskal-Wallis chi-squared = 7325.1, df = 6, p-value < 2.2e-16). (Significant level indicated by: 0.05 > p-value ≥ 0.01 = *, 0.01 > p-value ≥ 0.001 = **, 0.001 > p-value = ***). INS removed from analysis due to a sample size of 1.

Comparison	Z Test Statistic	Adjusted p-values	Sig.
SNPoutlier-SNPnonoutlier	0.350104	1.00E+00	
SNPoutlier-SVoutlier	-7.74876	1.48E-13	***
SNPoutlier-SVnonoutliersDEL	-9.12597	1.28E-18	***
SNPoutlier-SVnonoutliersDUP	-5.63893	2.40E-07	***
SNPoutlier-SVnonoutliersTRA	-7.09468	1.94E-11	***
SNPoutlier-SVnonoutliersINV	2.636103	7.55E-02	
SNPnonoutlier-SVoutlier	-14.3004	4.13E-45	***
SNPnonoutlier-SVnonoutliersDEL	-84.6074	0.00E+00	***
SNPnonoutlier-SVnonoutliersDUP	-8.35485	1.11E-15	***
SNPnonoutlier-SVnonoutliersTRA	-14.6467	2.83E-47	***
SNPnonoutlier-SVnonoutliersINV	2.61253	7.19E-02	
SVoutlier-SVnonoutliersDEL	0.273198	1.00E+00	
SVoutlier-SVnonoutliersDUP	1.18469	9.45E-01	
SVoutlier-SVnonoutliersTRA	1.289987	1.00E+00	
SVoutlier-SVnonoutliersINV	3.855428	1.50E-03	**
SVnonoutliersDEL-SVnonoutliersDUP	1.241671	1.00E+00	
SVnonoutliersDEL-SVnonoutliersTRA	1.641626	7.05E-01	
SVnonoutliersDEL-SVnonoutliersINV	3.845829	1.44E-03	**
SVnonoutliersDUP-SVnonoutliersTRA	-0.23646	8.13E-01	
SVnonoutliersDUP-SVnonoutliersINV	3.656451	2.56E-03	**
SVnonoutliersTRA-SVnonoutliersINV	3.711027	2.27E-03	**

REFERENCES:

Hofmeister NR, Stuart K, Warren WC, Werner SJ, Bateson M, Ball GF, Buchanan KL, Burt DW, Cardilini APA, Cassey P *et al.* 2021 Concurrent invasions by European starlings (Sturnus vulgaris) suggest selection on shared genomic regions even after genetic bottlenecks. 2021.05.19.442026. (doi:10.1101/2021.05.19.442026)

Rollins LA, Woolnough AP, Wilton AN, Sinclair R & Sherwin WB 2009 Invasive species can't cover their tracks: using microsatellites to assist management of starling (Sturnus vulgaris) populations in Western Australia. *Molecular Ecology* **18** 1560–1573. (doi:10.1111/j.1365-294X.2009.04132.x)

Stuart KC, Cardilini APA, Cassey P, Richardson MF, Sherwin WB, Rollins LA & Sherman CDH 2021 Signatures of selection in a recent invasion reveal adaptive divergence in a highly vagile invasive species. *Molecular Ecology* **30** 1419–1434. (doi:10.1111/mec.15601)
